# Impacts of ecosystem service message framing and dynamic social norms on public support for tropical forest restoration

**DOI:** 10.1111/cobi.14373

**Published:** 2024-09-10

**Authors:** D.‐L. Simons, R. B. Bradbury, K. L. Evans

**Affiliations:** ^1^ Ecology and Evolutionary Biology, School of Biosciences University of Sheffield Sheffield UK; ^2^ Department of Earth, Ocean and Ecological Sciences, School of Environmental Science University of Liverpool Liverpool UK; ^3^ RSPB Centre for Conservation Science Cambridge UK; ^4^ Conservation Science Group, Department of Zoology University of Cambridge Cambridge UK

**Keywords:** behavioral change, charitable fund‐raising, dynamic norm nudge, ecological restoration, nature conservation, cambio conductual, conservación de la naturaleza, empuje de normas dinámicas, recaudación de fondos, restauración ecológica

## Abstract

The effectiveness of strategic psychology‐based marketing techniques for increasing public support for conservation is poorly understood. We assessed how such techniques affect support for tropical rainforest restoration with a controlled online experiment with 1166 nationally representative residents of the United Kingdom. We tested whether support increased when adding ecosystem service (ES) framings to typical nongovernmental organizations’ (NGOs) biodiversity‐focused messages that emphasize benefits to UK residents or people living near the tropical restoration site and a dynamic social norm nudge that emphasized increasing popularity of environmental restoration. We considered how respondents’ psychological traits (nature connection, self‐efficacy, psychological benefits of supporting charities, awareness of environmental degradation in the Global South, and climate change skepticism) influenced responses. Outcomes included respondents’ reported advertisement sufficiency, sympathetic attitudes, behavioral support, and financial support. The study population typically found advertisements sufficient and exhibited sympathetic attitudes and financial, but not behavioral, support. Younger people exhibited greater conservation support than older respondents. Messages framed solely on biodiversity conservation were as effective as those highlighting additional ES benefits received by UK residents and people near the tropical restoration site. This suggests that framing around ESs, rather than nature's intrinsic value, may not strengthen public support for conservation. The dynamic social norm nudge had perverse effects. It reduced perceived social norms and most outcome variables. Alternative dynamic norm nudges warrant testing, but our results support research suggesting dynamic norm nudges can be ineffective when associated with activism, challenging their use by conservation NGOs. Psychological benefits of supporting charities and perceived self‐efficacy increased support for advertisements, highlighting the benefits of including impact statements relating respondents’ support to specific outcomes. Climate change skepticism decreased support, whereas nature connection and perceived static social norms increased it, highlighting the need to increase nature connection and pro‐environmental social norms to elevate public support for conservation.

## Graphical abstract

**The study**: Recorded responses of > 1000 UK adults towards different marketing strategies. Tested 6 treatments ‐ 3 message framing strategies in the presence and absence of a dynamic social norm nudge. Tested how respondents' psychological traits influence advert effectiveness.

**Findings**: Public were sympathetic and financially supportive towards conservation but did not show behavioral support. Message framing did not influence advert effectiveness. Dynamic social‐norm nudge adversely affected support which contrasted with positive effect of static social norms. Self‐efficacy, psychological benefits, and nature connection increased support. Climate change skepticism decreased support.

**Take homes**: Financial support easier to gain than behavioral support. Promote self‐efficacy and psychological benefits in campaigns. Enhance nature connection long‐term. Caution the use of dynamic‐norm nudges in conservation marketing. Ecosystem service messages may not increase public support for conservation.

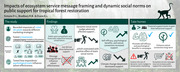

## Abstract (es)

Impactos del encuadre de los mensajes sobre servicios ambientales y las normas sociales dinámicas sobre el apoyo público hacia la restauración de bosques tropicales

**Resumen:**

Sabemos poco sobre la eficiencia de las técnicas de mercadotecnia basadas en la psicología estratégica para aumentar el apoyo público a la conservación. Evaluamos cómo afectan dichas técnicas al apoyo a la restauración de la selva tropical mediante un experimento controlado en línea con 1,166 residentes del Reino Unido representativos a nivel nacional. Comprobamos si el apoyo aumentaba al añadir marcos de servicios ambientales a los mensajes típicos de las organizaciones no gubernamentales (ONG) centrados en la biodiversidad, que hacen hincapié en los beneficios para los residentes del Reino Unido o las personas que viven cerca del lugar de restauración tropical y un empuje dinámico de normas sociales que hacía hincapié en la creciente popularidad de la restauración ecológica. Analizamos la influencia de los rasgos psicológicos de los encuestados (conexión con la naturaleza, autoeficacia, beneficios psicológicos de apoyar a organizaciones benéficas, experiencia de degradación ambiental en el Sur Global y escepticismo ante el cambio climático) sobre las respuestas. Los resultados fueron la suficiencia de los anuncios, las actitudes de simpatía, el apoyo conductual y el apoyo económico. En general, la población del estudio consideró que los anuncios eran suficientes y mostró actitudes de simpatía y apoyo económico, pero no conductuales. La población más joven mostró un mayor apoyo a la conservación que los encuestados de más edad. Los mensajes centrados únicamente en la conservación de la biodiversidad fueron tan eficaces como los que destacaban los beneficios adicionales de los servicios ambientales recibidos por los residentes del Reino Unido y las personas cercanas al lugar de restauración tropical. Esto sugiere que el encuadre en torno a los servicios ambientales, en lugar del valor intrínseco de la naturaleza, puede no reforzar el apoyo público a la conservación. El empuje dinámico de la norma social tuvo efectos perversos ya que redujo las normas sociales percibidas y la mayoría de las variables de resultado. Es necesario probar otros incentivos dinámicos, pero nuestros resultados corroboran las investigaciones que sugieren que los incentivos dinámicos pueden ser ineficaces cuando se asocian con el activismo, lo que cuestiona su uso por parte de las ONG de la conservación. Los beneficios psicológicos por apoyar a organizaciones benéficas y la autoeficacia percibida aumentaron el apoyo a los anuncios, lo que resalta las ventajas de incluir declaraciones de impacto que relacionen el apoyo de los encuestados con resultados específicos. El escepticismo ante el cambio climático redujo el apoyo, mientras que la conexión con la naturaleza y las normas sociales estáticas percibidas lo aumentaron, lo que destaca la necesidad de aumentar la conexión con la naturaleza y las normas sociales proambientales para elevar el apoyo público a la conservación.

## INTRODUCTION

Conservation relies heavily on public donations (WWF UK, 2022) and volunteering (Balmford et al., 2021) via environmental nongovernmental organizations (ENGOs), but financial and behavioral support remains insufficient (Anyango‐van Zwieten et al., 2019; Ockwell et al., 2009). Despite long‐standing criticism that poor design of conservation campaigns limits their effectiveness (Simmons, 1998), campaign strategy evaluation remains rare and of questionable quality (Olmedo et al., 2020). Strategic psychology‐based techniques can improve marketing designs with little or no additional cost (Kusmanoff et al., 2020; Nielsen et al., 2021; Wright et al., 2015). These techniques include altering messages’ rationale or structure (i.e., message framing) (Kamenica, 2012); nudging tools that influence automatic, unconscious cognitive processes (Byerly et al., 2018); and targeting audience segments (e.g., psychological traits of message recipients) (Slater, 1996). Research testing these techniques in conservation marketing to promote pro‐environmental behavior, particularly when applied in combination, is limited (Balmford et al., 2021; Ryan et al., 2019).

Audience segmentation is frequently used by marketers (Slater, 1996) and has proven effective in contexts of climate change (Hine et al., 2014) and conservation (Jones et al., 2019). Considering audience segments when designing campaigns allows resource optimization by targeting groups most likely to respond positively to interventions. Examples of audience segments important to intervention success include an individual's perceived ability to make a difference (i.e., self‐efficacy; e.g., believing a donation will generate positive change; Ryan & Deci, 2000), psychological benefits gained by engaging in charitable activities (e.g., increased happiness) (Andreoni, 1990; Dunn et al., 2008), and broader sociodemographic traits. Examining audience segmentation alongside the effectiveness of different interventions, such as message framing and nudges, enables the identification of distinct psychological processes that influence support for conservation (Loschelder et al., 2019).

Although message framing can strongly influence individual attitudes (Kidd et al., 2019; Kusmanoff et al., 2016), strategy success can relate to motivational differences between audience segments (Schultz, 2001; Weinstein et al., 2015). Messages promoting the intrinsic value of conserving biodiversity successfully target individuals with high biospheric attitudes (Helm et al., 2018; van der Werff et al., 2013) and strong connection to nature (Whitburn et al., 2020; Zylstra et al., 2014). However, solely focusing on biodiversity can fail to attract egotistically motivated individuals (Kusmanoff et al., 2016) and individuals who prioritize people over nature. An alternative utilitarian approach, now often adopted by ENGOs alongside biodiversity‐focused messages, is to frame messages around human benefits by emphasizing the effects of conservation on ecosystem services (ESs) (Martín‐López et al., 2012). Messages that highlight ES benefits of conservation will likely attract individuals who understand the links between environmental degradation and ES provision (McDonald et al., 2015). Considerable debate surrounds the merits of biodiversity versus ES approaches (Bekessy et al., 2018; Mace, 2014), with recent empirical evidence suggesting that message framing has limited influence on donation behavior for conservation campaigns (Blake et al., 2023; Shreedhar, 2021). However, advertisements that highlight combinations of biodiversity and ES framing could theoretically provide a more holistic portrayal of conservation values (Matzek & Wilson, 2021). They could elicit support from individuals with a wide range of values and thus increase overall support compared with strategies that reflect the traditional ENGO approach of only highlighting biodiversity benefits.

Alternative approaches to ES framings can attract different audience segments (Opdam et al., 2015). People in the Global North, where most ENGOs are based, commonly prioritize donations that support vulnerable people over conservation (Coldwell & Evans, 2017). Therefore, to gain support from this demographic for conservation, framing could emphasize local benefits for disadvantaged communities, attracting altruistic individuals (Lu & Schuldt, 2016). Alternatively, framing around global benefits that also accrue to individuals in the Global North, such as climate change mitigation, could attract egotistic individuals (Sapiains et al., 2016; Scannell & Gifford, 2013). Empirically comparing different ES framings, such as those focused on local versus global scales, will offer further insights into the nuanced effects of framing on decision‐making.

In addition to message framing, nudges can effectively promote pro‐environmental behavior (Byerly et al., 2018; Thaler & Sunstein, 2021). Specifically, descriptive social norm nudges (i.e., interventions describing the frequency of shared actions within social groups) reliably shift behaviors across multiple domains, including public administration (John et al., 2014) and environmental change (Cialdini & Jacobson, 2021; Farrow et al., 2017). However, the use of descriptive norms to shift behavioral patterns is not universally successful, depending on the ease of compliance and competing messaging from other reference groups (John et al., 2014). Within biodiversity conservation, dynamic norms, which emphasize shifts in behaviors over time, could be more suitable than static norms because many pro‐environmental behaviors do not yet dominate in the Global North. In such circumstances, dynamic norms can better adapt to evolving social behaviors and environmental circumstances than static norms and are more effective at generating sustained behavioral change (Loschelder et al., 2019). Dynamic norms have effectively shifted real‐world behaviors within the domains of diet (Sparkman & Walton, 2017), waste disposal (Loschelder et al., 2019), water conservation (Mortensen et al., 2019), and climate policy (Sabherwal et al., 2021). However, robust empirical evaluations that incorporate descriptive dynamic norms into conservation marketing are needed to assess their context‐specific effectiveness, as well as any potential perverse and unanticipated impacts (Chung & Rimal, 2016; Perry et al., 2021).

We conducted an online experiment to address 3 core research questions regarding conservation marketing material designed to elicit public support from the Global North for tropical rainforest restoration. First, we tested whether additional ES framings increased the effectiveness of conservation advertisements with traditional biodiversity framing. Second, we compared the effectiveness of 2 differing ES framings: local‐scale ESs that benefit vulnerable people in the Global South living near the restoration site and global‐scale ESs that benefit the responder living in the Global North. Finally, we tested whether the effectiveness of each message framing was altered by incorporating a descriptive dynamic social norm nudge. We also assessed how support is associated with respondents’ psychological traits, including perceived self‐efficacy, receipt of psychological benefits from supporting charities, nature connection, awareness of degradation in the Global South, and climate change skepticism.

## METHODS

Our study design was approved by the University of Sheffield's Research Ethics Committee in March 2020 (reference 032876) and tested in a pilot study (see Appendix S1). To promote reproducibility, all data, scripts, and figures for the manuscript are available in the Git repository (https://github.com/dinaleighsimons/Simons‐Bradbury‐Evans_24).

### Respondent recruitment

A power analysis for linear multiple regression was conducted in the package pwr in R (R Core Team, 2024). This concluded that a minimum sample size of 1042 was required (174 per treatment) to give a large power (β = 0.8) for models (whose covariates had the intended df of 19) to detect a statistically significant (α = 0.05) small effect size (Cohen's *f* ^2^ = 0.02 obtained with the function cohen.ES based on estimates from Cohen [1988]). The conservative choice of a small effect size ensured sensitivity to detect realistic effects, given the lack of comparable studies at the time of data collection and the small effects that are often detected in psychological research (Lovakov & Agadullina, 2021).

A nationally representative sample tool (based on age, gender, and ethnicity) was used to recruit a target of 1100 adult UK residents from the online platform Prolific (https://prolific.co/) in April 2020. Prolific conducts prescreening to allow unbiased enrollment of respondents (Palan & Schitter, 2018). Respondents were paid £5 per hour (pro rata, in accordance with Prolific's protocol at the time).

### Treatments

**FIGURE 1:**
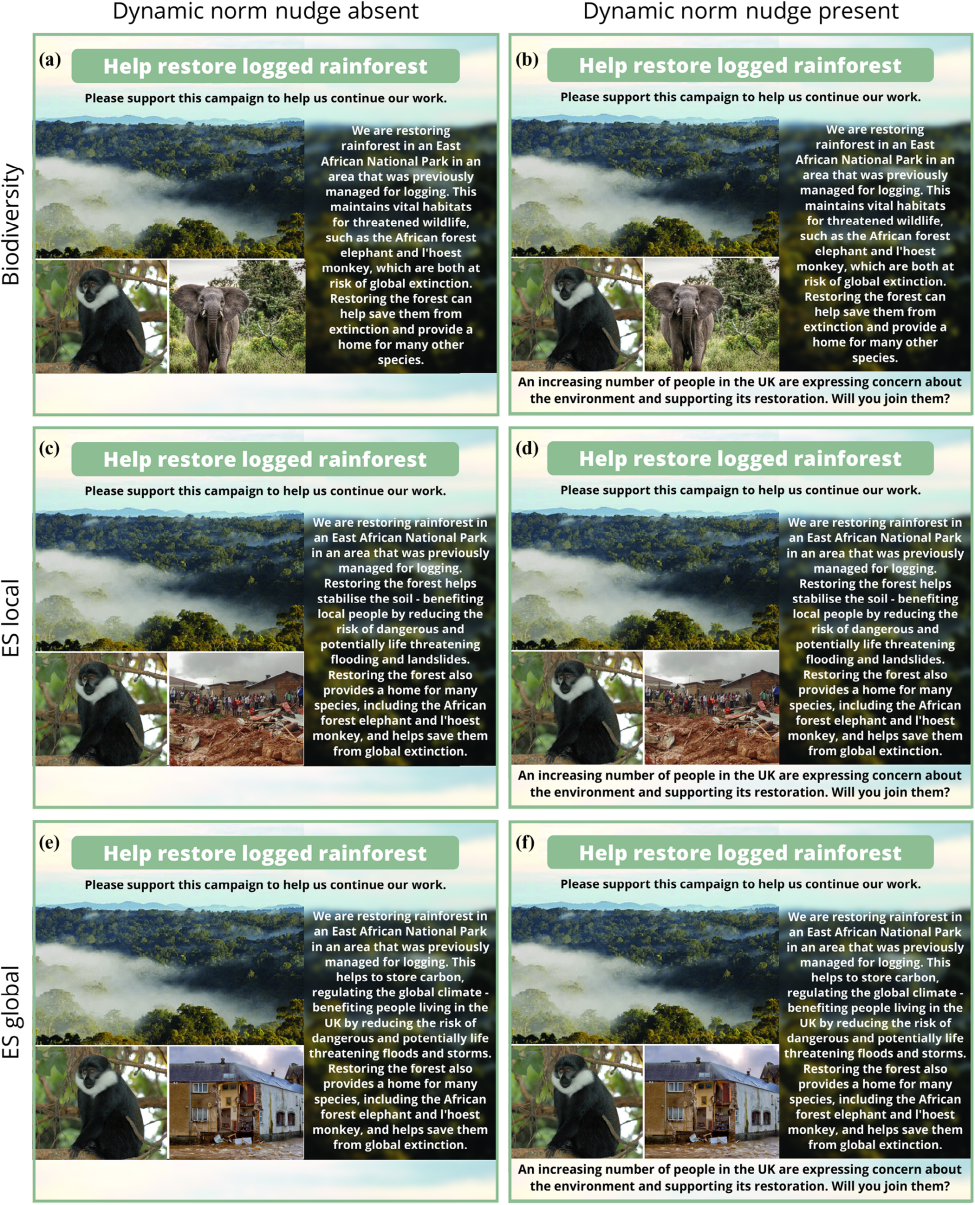
Experimental design showing 6 treatments with (a) biodiversity, (b) biodiversity with dynamic norm nudge, (c) biodiversity + local ecosystem services (ESs), (d) biodiversity + local ESs with dynamic norm nudge, (e) biodiversity + global ESs, and (f) biodiversity + global ESs with dynamic norm nudge. The species illustrated to draw attention to conservation targets (L'Hoest's monkey [*Allochrocebus lhoesti*] and African elephant [*Loxodonta africana*]) are widely used as conservation targets due to their charismatic and aesthetic appeal (Smith et al., 2012; Wolf & Ripple, 2022).

Respondents were randomly allocated one of 6 advertisements for a tropical rainforest restoration campaign in a region affected by environmental degradation (Figure 1). The allocation process was double blinded. The campaign was based on a case study of logged forest restoration in Kibale National Park (Uganda, East Africa) to reflect the crucial role of tropical forest restoration for global conservation and climate change mitigation (Erbaugh et al., 2020; Mackenzie & Ahabyona, 2012). The case study location was not specific in advertisements to minimize bias from respondents seeking additional information about the location that might influence their response.

We designed controlled advertisements, consistent with typical social media infographics, that contained realistic aesthetic elements based on similar designs from related studies (Kubo et al., 2023; Marquina et al., 2022) (Figure 1). Advertisement text and secondary images varied among treatments to reflect different message framings. The treatment design, including the primary image, headline, length of text (60–73 words), and the request for support, was similar.

We used 3 message framing treatments: biodiversity (BD) (benefits for species), which was the control (Figure 1a,b); biodiversity + local‐scale ESs (BD+ES‐L) (Figure 1c,d) (benefits for people near restoration); and biodiversity + global‐scale ESs (BD+ES‐G) (Figure 1e,f) (benefits for UK respondents). Our control reflected the default traditional biodiversity messaging of conservation‐focused ENGOs (Campos et al., 2021), to which ES‐focused messages are sometimes added. Both local and global ES framings emphasized regulation of potentially life‐threatening flooding events. Global ESs focused on mitigating climate change via carbon storage, and local ESs focused on restoring local forest cover to stabilize soil and prevent landslides. We predicted that the addition of ES framings would increase overall support relative to biodiversity control (Kusmanoff et al., 2020; Matzek & Wilson, 2021).

To test whether nudging tools influenced support, we created 2 versions of the 3 framing treatments that included or excluded a descriptive dynamic social norm nudge (henceforth referred to as dynamic norm nudge) for a total of 6 treatments (Figure 1). The text read, “An increasing number of people in the UK are expressing concern about the environment and supporting its restoration. Will you join them?” (Figure 1b,d,f). We predicted that this dynamic norm nudge would increase support across all message framing treatments.

The full questionnaire (Appendices S3 & S4), hosted via Survey Monkey (https://www.surveymonkey.com/), was designed to ensure compatibility with mobile devices. Each respondent randomly received one treatment and could only complete the survey once, which reduced the risk of familiarity or learning effects influencing results (Manahova et al., 2020). Each treatment was allocated to 16.67% of the respondent pool, equalizing sample size across treatments. To test attention, respondents were shown a letter (A–F) corresponding to the treatment (i.e., advertisement) allocated to them and subsequently asked to state that letter (see “Data analyses”).

### Measuring responses

Questions were designed to elicit quantitative responses through continuous slider scales, which increases engagement and accuracy (DeCastellarnau, 2018; Roster et al., 2015). Sliders were predominately on a 21‐point scale, with descriptive labels at the extremes and center of the slider (0 = *strongly disagree/extremely unlikely*, 10 = *neither agree nor disagree/neither likely nor unlikely*, and 20 = *strongly agree/extremely likely*). This scale, relative to larger ranges, prevented respondents from being overwhelmed with choices that could cause bias (Liu & Conrad, 2019). To enhance user experience, an interactive numeric box next to the slider showed respondents their chosen value. The slider button was initially set in a neutral position, with it and the numeric box grayed out, to prevent starting position bias (Funke, 2016). To retain respondent engagement, 6 out of 23 slider scale questions were reverse coded, so higher values related to more negative responses. These were recoded prior to analysis. The only quantitatively measured questions not to use the 21‐point slider scale had preexisting scales (i.e., the nature relatedness scale [Nisbet & Zelenski, 2013] and flood experience [McLeod, 2014]) (full descriptions in “Predictor variables”).

### Outcome variables

We recorded advertisement sufficiency (i.e., whether the advertisement provided adequate information) through a quantitatively measured statement, “The advert alone gave me enough information to decide whether to support this campaign.”

We quantified sympathetic attitude through a metric derived from principal component analysis (PCA) of responses to 2 statements: “I felt supportive towards this advert” and “I hope that this campaign for rainforest restoration succeeds.” Linear PCA, following the standardization of responses to have a mean of zero and variance of one, was conducted in R with the function prcomp (R Core Team, 2024). All following PCAs used the same method. The PCA variables loaded onto a single axis (eigenvalue = 1.67; component loadings: supportive −0.71, success −0.71; variance explained = 83.19%).

Behavioral support was measured with a PCA‐derived metric of 5 quantitatively measured questions. These captured respondents’ willingness to sign petitions, promote the campaign via conversations and social media, sacrifice time, and purchase products contributing to the destruction of the focal rainforest (questions in Appendix S4). The PCA variables loaded onto a single axis (eigenvalue = 2.76; component loadings: petition −0.46, conversation −0.52, social media −0.50, products −0.13, time −0.49; variance explained = 55.28%). All other PCA axes had eigenvalues <1, so we used PCA scores extracted from the first dimension as a measure of behavioral support.

Financial support was measured with a PCA‐derived metric of 2 quantitatively measured questions. Respondents reported their donation size (“How many pounds [£] would you be willing to give as a one‐off donation to this campaign?”) and the proportion of £100 they would allocate to the campaign (“If you were given £100 to support any charitable activity, how much would you allocate to support the advert's campaign?”). This approach was designed to capture 2 self‐reported measures of willingness to donate, including and excluding personal financial constraints. The PCA variables loaded onto a single axis (eigenvalue = 1.31; component loadings: donation size 0.71, proportion allocated 0.71; variance explained = 65.55%).

### Predictor variables

Both ES framings reflected how supporting ecological restoration could mitigate flooding (Figure 1). Thus, we measured respondents’ personal experiences of adverse flooding impacts, adapting the methods of McLeod (2014). We asked how many times in the last 5 years had floods caused significant changes to their daily routine, difficulty accessing their residence, and damages to, or loss of, their possessions. Each question was measured on a 7‐point scale representing the number of flood events experienced (0, 1, 2, 3–5, 6–10, 11–15, and more than 15) (Appendix S4). To create a single continuous flood experience variable, we assigned a midpoint value to each bounded category (e.g., 4 for category 3–5) and calculated the average response across the 3 questions. For the uppermost unbounded category (more than 15), which was chosen by 13 respondents (0.01% of our sample size), we allocated a midpoint of 17. We anticipated that greater flood experience would increase support for both ES framings relative to the biodiversity framing.

We asked questions to test a series of secondary hypotheses, that respondents would support a campaign more if they had a greater connection to nature, greater perceived self‐efficacy, psychological benefits from supporting charities, experience of the Global South, and less skepticism toward climate change. The short‐form nature relatedness scale was used to measure respondents’ connection to nature, and responses were averaged to create a continuous variable, as is standard for this scale (Nisbet & Zelenski 2013) (questions in Appendix S4). Responses were measured on a 7‐point Likert scale (increased from 5 points to gain a wider range of values), where *strongly disagree* equated to 0 and *strongly agree* equated to 7.

Self‐efficacy was recorded by one quantitatively measured question (“I believe that if I supported this campaign, it would make an impactful difference.”). Our measure for psychological benefits of providing support was a PCA‐derived metric of 2 quantitatively measured questions, from Dickert et al. (2011) (“Supporting a charitable cause makes me feel better in myself” and “I often feel regret or guilt if I don't support a charitable cause after being asked to.”) The PCA variables loaded onto a single axis (eigenvalue = 1.49; component loadings: feeling better 0.71, guilt 0.71; variance explained = 74.41%). We anticipated that self‐efficacy and psychological benefits would predict increased support.

Support for conservation in the Global South could be linked to respondents’ awareness of such environments, especially environmental degradation, and its impacts on local people. We quantified awareness of the Global South with a PCA‐derived metric of 3 quantitatively measured questions: “I have personally witnessed in real life the adversity people face in poor, developing countries”; “I have personally witnessed through television, online or local campaigns, the adversity people face in poor, developing countries”; and “I believe people are suffering in Africa because of environmental degradation caused by human activities.”). The PCA variables loaded onto a single axis (eigenvalue = 1.49; component loadings: real life 0.51, virtual 0.65, human activities 0.57; variance explained = 49.57%). Other PCA axes had eigenvalues lower than 1.

Questions used to measure climate change skepticism were adapted from Whitmarsh (2011) and asked how much respondents agreed with claims that human activities changing the climate are exaggerated, that climate change is just a natural fluctuation in Earth's temperatures, and that climate change is not a real problem (reverse‐coded); high values represented more skepticism. The PCA variables loaded onto a single axis (eigenvalue = 2.41; component loadings: exaggerated −0.58, fluctuation −0.58, real problem −0.57; variance explained = 80.17%). Other PCA axes had eigenvalues <1. We anticipated that climate change skepticism would lower support for ecological restoration.

Two additional social norm measures were used to check the manipulation capability of the dynamic norm nudge treatment and determine how perceived social norms affect support (i.e., a respondent believed their response matched preexisting social norms). Our measure of social norm support was a PCA‐derived metric of 2 quantitatively measured questions “Most other members of the public would hope that this campaign succeeds” and “Most other members of the public would support this campaign”. The PCA variables loaded onto a single axis (eigenvalue = 1.58; component loadings: succeed −0.71, support −0.71; variance explained = 79.20%). Social norm donation was recorded by one quantitatively measured question “On average, how many pounds (£) do you think other people would donate as a one‐off‐donation to this campaign?” We anticipated that the dynamic norm nudge would increase support for the campaign and that those perceiving strong social norms (i.e., believing others would support the campaign) would show increased support.

### Sociodemographics

Sociodemographic factors were included as predictors in models to increase robustness when testing our focal variables. These included financial security, age, gender, index of multiple deprivation (IMD), education, and ethnicity (see Appendix S2 for how factors were derived).

### Data analyses

We received 1235 responses. Following best practice (Leiner, 2019; Maniaci & Rogge, 2014), we removed data from 69 inattentive respondents, who were distributed across all 6 treatments. Inattentive respondents were removed for one of 4 reasons. First, they failed the attention test described above (*n* = 1). Second, their self‐reported donation was unrealistic given their financial security score (*n* = 3) (i.e., donating £1000 or more when their financial security score was extremely low, <15). Third, incomplete postcodes were provided, which prevented us from obtaining their IMD (*n* = 32). Finally, they gave incomplete responses (*n* = 33). Our final sample size (1166; Table 1) exceeded the required sample size identified with the power analysis.

**TABLE 1:** Sample sizes of adult UK respondents (following removal of inattentive respondents) per treatment in the experimental survey that measured support for different conservation advertisements.

	Biodiversity	Biodiversity + local scale ecosystem services	Biodiversity + global scale ecosystem services	Total
Dynamic norm nudge absent	197	193	189	579
Dynamic norm nudge present	219	186	182	587
Total	416	379	371	1166

For advertisement sufficiency, sympathetic attitudes, and behavioral support, we used one‐sample *t*‐tests to assess whether responses differed from PCA scores equating to neither agree nor disagree on the 21‐point scale. For financial support, we used one‐sampled Wilcoxon signed rank tests to assess whether the self‐reported amount of money donated and the proportion of the hypothetical £100 donated to the campaign were significantly >0.

Full multiple regression models were constructed for each outcome variable following Whittingham et al. (2006) with core functions in RStudio 4.3.1 (R Core Team, 2024). Multicollinearity was checked with the variance inflation factor (VIF), calculated in the package performance in RStudio. In all cases, VIF values were consistently <2. This is substantially below the threshold value of 10, above which collinearity can distort the interpretation of multiple regression models (Dormann et al., 2013). In all regression models, we conducted type II analysis of variance (ANOVA) with an *F* test to test significance in the package car (Fox & Weisberg, 2018). For all constructed models, model assumptions (linearity, normality, constant variance, and leverage) were checked using diagnostic plots created through the autoplot() function in the package ggfortify (Tang et al., 2016); all assumptions were sufficiently met.

To test whether the dynamic norm nudge manipulated respondents’ perceptions of social norms, we modeled perceived social norm support in a general linear model framework as a function of dynamic norm nudge (2‐level categorical variable), financial security (continuous), age (continuous), gender (3‐level categorical variable: female, male, and other), IMD (continuous), education (continuous), and ethnicity (2‐level categorical variable: white and other; finer subdivision was not feasible due to small numbers of respondents in alternative categories). The same model structure was used for the social norm donation metric (natural log transformed). Because dynamic norm messages can increase perceptions of self‐efficacy (Sparkman & Walton, 2019), the same model structure was used to model self‐efficacy (continuous).

Advertisement sufficiency was modeled in a general linear model framework, as a function of message framing (3‐level categorical variable) and dynamic norm nudge while accounting for self‐efficacy, nature connection (continuous), psychological benefits (continuous), Global South awareness (continuous), climate change skepticism (continuous), flood experience (continuous), IMD, financial security, education, gender, age, and ethnicity. Social norm measures (support and donation) were not included in this model because there was no clear causal pathway through which they could influence respondents’ perceptions of advertisement sufficiency. The dynamic norm nudge was included because additional information in the nudge treatments could influence respondents’ opinions of sufficiency. The same model structure was used to model sympathetic attitudes and behavioral support, with the inclusion of social norm support (continuous) as an additional predictor.

Financial support was modeled with a generalized linear model with a quasi‐Poisson distribution and log link function, and the following predictors: message framing, dynamic norm nudge, self‐efficacy, nature connection, psychological benefits, Global South awareness, social norm donation (natural log transformed; selected rather than social norm support to better match the response variable), climate change skepticism, flood experience, IMD, financial security, education, gender, age, and ethnicity.

Although our study was underpowered to test for interactions between treatments and audience segmentation variables, we provide exploratory results of these relationships in Appendices S9 and S10. For each outcome variable, we constructed a full model containing only the main effects and proceeded to test the significance of each interaction. To account for multiple comparisons, we controlled for false discovery rate (FDR) (Benjamini & Hochberg, 1995) with the R function p.adjust(). The FDR adjustments did not change our core results, except in one case (see “Results”). Unadjusted *p* values are reported in “Results,” and adjusted *p* values are reported in Appendices S8–S10.

## RESULTS

### Sample sociodemographics and dynamic norm nudge manipulation checks

Across treatments, respondents represented the UK population in terms of age, gender, and ethnicity, albeit with a minimal overrepresentation of age 55–64 and an underrepresentation of age 75+ (Appendix S5). Respondents’ attributes (self‐efficacy, nature connection, Global South awareness, climate change skepticism, flood experience, IMD, financial security, education, and age) were consistent across treatments (Appendix S6).

The dynamic norm nudge, when considering respondents’ attributes, decreased perceived social norm support (*F*_1, 1157_ = 14.4, *p* = 0.0002) but did not influence social norm donation (*F*_1, 1157_ = 1.1, *p* = 0.29) or self‐efficacy (*F*_1, 1157_ = 3.2, *p* = 0.072) (Appendix S7).

### Advertisement sufficiency

Advertisement sufficiency of respondents on the original 21‐point Likert scale (mean [SE] = 12.09 [0.16]) was significantly higher than the score equating to neither agree nor disagree (i.e., 10; *t* = 13.1, df = 1165, *p* = 2.2 × 10^−16^).

**FIGURE 2:**
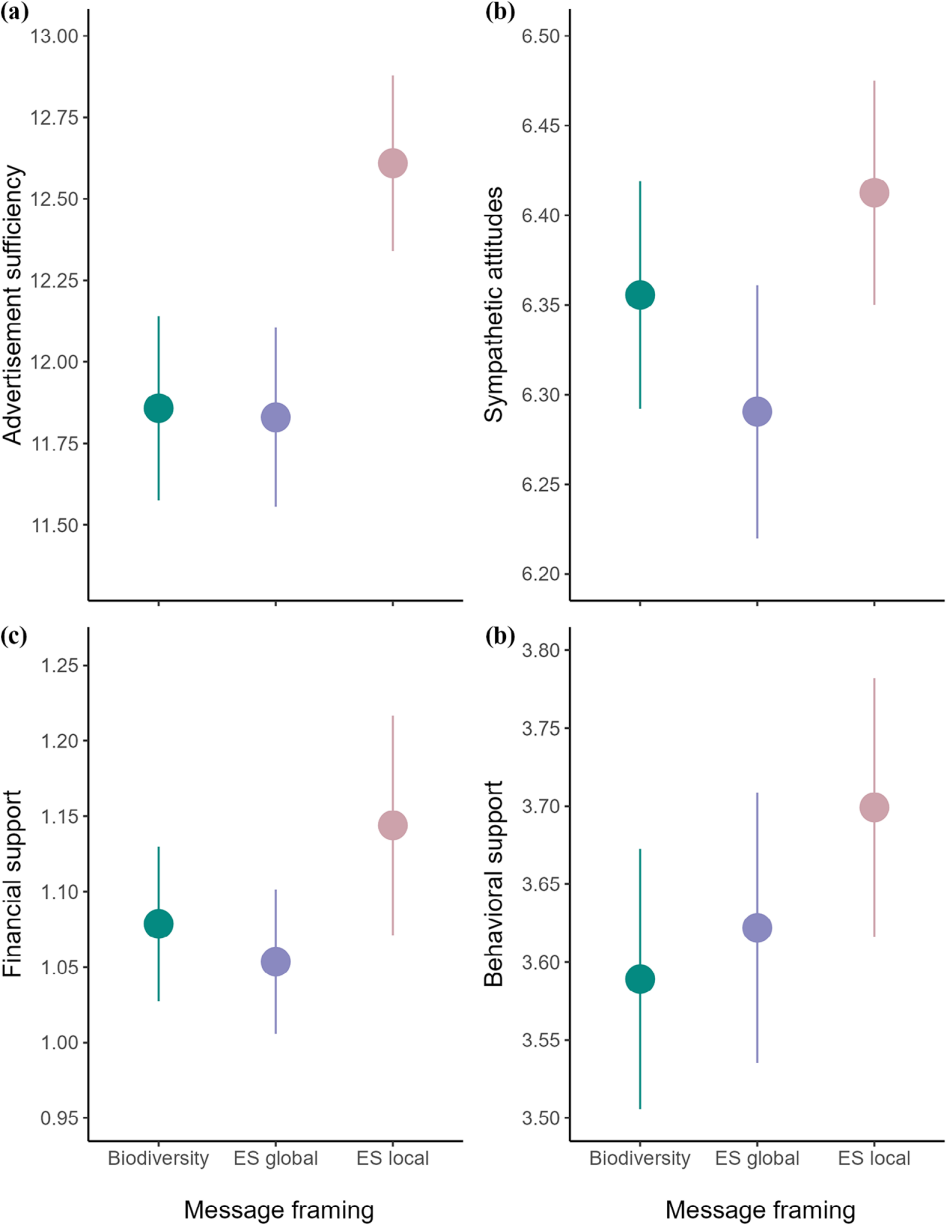
The mean (SE) of the response variables (a) advertisement sufficiency, (b) sympathetic attitudes, (c) financial support, and (d) behavioral support of respondents exposed to advertisements with biodiversity, global ecosystem service (ES global), or local ES (ES local) message framing (see Figure 1). Advertisement sufficiency based on one question on a 21‐point Likert scale (0 = *strongly disagree/highly unlikely*; 20 = *strongly agree/highly likely*). Other response variables are principal component analysis scores from multiple questions: sympathetic attitudes, score of 6 is a Likert score of ∼15 on the 21‐point Likert scale for all questions; behavioral support, score of 3.6 is a Likert score of ∼10; financial support, score of 1.0 is reported donation of ∼£30 of a hypothetical £100 available to give to campaign charities.

**FIGURE 3:**
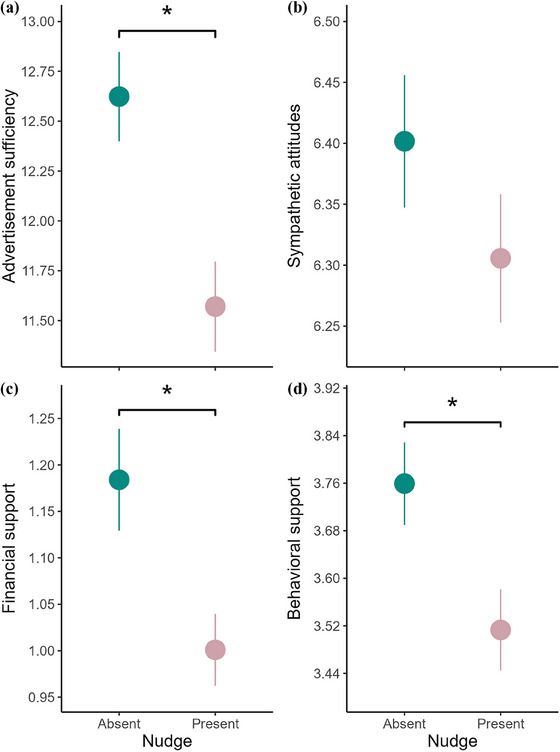
The mean (SE) of the response variables (a) advertisement sufficiency, (b) sympathetic attitudes, (c) financial support, and (d) behavioral support of respondents exposed to advertisements with and without the dynamic norm nudge (asterisk, significance at α = 0.05). See the legend of Figure 2 for more information on the response variables.

Message framing did not affect advertisement sufficiency (Table 2; Figure 2a). Advertisements containing the dynamic norm nudge showed a significant decrease in advertisement sufficiency compared with the control (Table 2; Figure 3a). Psychological factors positively associated with sufficiency were self‐efficacy and psychological benefits (Table 2). Education level was negatively associated with sufficiency, and older people found the advertisements’ information more sufficient (Table 2).

**TABLE 2:** Parameter estimates from the full multiple regression models of the 4 outcome variables (advertisement sufficiency, sympathetic values, financial support, and behavioral support) used to measure support for conservation advertisements through quantitative survey questions.

Predictor	Level	Advertisement sufficiency (SE)	Sympathetic attitude (SE)	Financial support (SE)	Behavioral support (SE)
Message framing (relative to BD)	BD + ES‐L	0.500 (0.354) *F*_2, 1149_ = 2.3, *p* = 0.158	0.013 (0.070) *F*_2, 1148_ = 1.0, *p* = 0.853	0.012 (0.060) *F*_2, 1148_ = 0.1, *p* = 0.838	−0.012 (0.087) *F*_2, 1148_ = 0.04, *p* = 0.889
	BD + ES‐G	−0.275 (0.356) *F*_2, 1149_ = 2.3, *p* = 0.441	−0.083 (0.071) *F*_2, 1148_ = 1.0, *p* = 0.239	−0.017 (0.062) *F*_2, 1148_ = 0.1, *p* = 0.779	−0.024 (0.087) *F*_2, 1148_ = 0.04, *p* = 0.784
Dynamic norm nudge (relative to absent)	Present	−0.703 (0.294) *F*_1, 1149_ = 5.7, *p* = 0.017^*^	−0.016 (0.058) *F*_1, 1148_ = 0.08, *p* = 0.781	−0.138 (0.050) *F*_1, 1148_ = 7.5, *p* = 0.006^*^	−0.150 (0.072) *F*_1, 1148_ = 4.3, *p* = 0.039^*^
Self‐efficacy		0.324 (0.035) *F*_1, 1149_ = 87.3, *p* = 2 × 10^−16*^	0.054 (0.007) *F*_1, 1148_ = 56.2, *p* = 1.3 × 10^−13*^	0.055 (0.006) *F*_1, 1148_ = 73.7*, p* = 2 × 10^−16*^	0.115 (0.009) *F*_1, 1148_ = 163.8, *p* = 2 × 10^−16*^
Nature connection		0.112 (0.153) *F*_1, 1149_ = 0.5, *p* = 0.464	0.210 (0.030) *F*_1, 1148_ = 47.6, *p* = 8.6 × 10^−12*^	0.169 (0.028) *F*_1, 1148_ = 37.8*, p* = 1.8 × 10^−9*^	0.370 (0.038) *F*_1, 1148_ = 96.5*, p* = 2 × 10^−16*^
Psychological benefits		0.594 (0.144) *F*_1, 1149_ = 17.0, *p* = 3.9 × 10^−5*^	0.089 (0.029) *F*_1, 1148_ = 9.7, *p* = 0.002^*^	−0.005 (0.026) *F*_1, 1148_ = 0.04, *p* = 0.835	0.184 (0.036) *F*_1, 1148_ = 26.9, *p* = 2.5 × 10^−7*^
Awareness of Global South		−0.005 (0.140) *F*_1, 1149_ = 0.001, *p* = 0.974	0.082 (0.028) *F*_1, 1148_ = 8.8, *p* = 0.003^*^	0.039 (0.025) *F*_1, 1148_ = 2.5, *p* = 0.116	0.055 (0.034) *F*_1, 1148_ = 2.6, *p* = 0.110
Social norm support		n/a	0.250 (0.026) *F*_1, 1148_ = 90.3, *p* = 2 × 10^−16*^	n/a	0.140 (0.033) *F*_1, 1148_ = 18.6, *p* = 1.8 × 10^−5*^
Social norm donation		n/a	n/a	0.281 (0.024) *F*_1, 1148_ = 122.6, *p* = 2 × 10^−16*^	n/a
Climate change skepticism		0.185 (0.105) *F*_1, 1149_ = 3.1, *p* = 0.078	−0.191 (0.021) *F*_1, 1148_ = 81.4, *p* = 2 × 10^−16*^	−0.030 (0.020) *F*_1, 1148_ = 2.5, *p* = 0.120	−0.090 (0.026) *F*_1, 1148_ = 12.2, *p* = 5 × 10^−4*^
Flood experience		0.029 (0.145) *F*_1, 1149_ = 0.04, *p* = 0.844	−0.067 (0.029) *F*_1, 1148_ = 5.4, *p* = 0.021^*^	−0.023 (0.023) *F*_1, 1148_ = 1.1, *p* = 0.315	0.056 (0.036) *F*_1, 1148_ = 2.5, *p* = 0.114
IMD		−0.053 (0.054) *F*_1, 1149_ = 1.0, *p* = 0.322	0.018 (0.011) *F*_1, 1148_ = 3.0, *p* = 0.085	−0.010 (0.009) *F*_1, 1148_ = 1.0, *p* = 0.316	−0.031 (0.013) *F*_1, 1148_ = 5.4, *p* = 0.021^*^
Financial security		0.010 (0.030) *F*_1, 1149_ = 0.1, *p* = 0.731	0.002 (0.006) *F*_1, 1148_ = 0.1, *p* = 0.710	−0.001 (0.005) *F*_1, 1148_ = 0.02, *p* = 0.875	−0.009 (0.007) *F*_1, 1148_ = 1.4, *p* = 0.235
Education		−0.735 (0.156) *F*_1, 1149_ = 21.8, *p* = 3.4 × 10^−6*^	−0.063 (0.031) *F*_1, 1148_ = 4.0, *p* = 0.045^*^	−0.010 (0.027) *F*_1, 1148_ = 0.1, *p* = 0.713	−0.053 (0.039) *F*_1, 1148_ = 1.9, *p* = 0.169
Gender (relative to female)	Male	0.043 (0.301) *F*_2, 1149_ = 0.8, *p* = 0.888	0.034 (0.060) *F*_2, 1148_ = 0.3, *p* = 0.568	−0.004 (0.052) *F*_2, 1148_ = 0.03, *p* = 0.943	−0.225 (0.074) *F*_2, 1148_ = 4.9, *p* = 0.002^*^
	Other	−2.755 (2.264) *F*_2, 1149_ = 0.8, *p* = 0.224	0.277 (0.449) *F*_2, 1148_ = 0.3, *p* = 0.537	0.104 (0.425) *F*_2, 1148_ = 0.03, *p* = 0.806	0.269 (0.556) *F*_2, 1148_ = 4.9, *p* = 0.628
Age		0.040 (0.010) *F*_1, 1149_ = 16.2, *p* = 6 × 10^−5*^	−0.006 (0.002) *F*_1, 1148_ = 8.1, *p* = 0.005^*^	−0.007 (0.002) *F*_1, 1148_ = 20.3, *p* = 7.3 × 10^−6*^	−0.008 (0.002) *F*_1, 1148_ = 9.5*, p* = 0.002^*^
Ethnicity (relative to white)	Other	0.427 (0.437) *F*_1, 1149_ = 1.0, *p* = 0.329	−0.159 (0.087) *F*_1, 1148_ = 3.4, *p* = 0.067	−0.135 (0.072) *F*_1, 1148_ = 3.6, *p* = 0.061	0.129 (0.107) *F*_1, 1148_ = 1.4*, p* = 0.230
*R* ^2 /^ *D* ^2^		*R* ^2^ = 0.18	*R* ^2^ = 0.43	*D* ^2^ = 0.31	*R* ^2^ = 0.47
df		16	17	17	17
*F*		5.72^*^	50.19^*^	26.72^*^	60.11^*^

Abbreviations: BD, biodiversity; ES‐G, global ecosystem services; ES‐L, local ecosystem services.

*Statistically significant.

### Sympathetic attitudes

Sympathetic attitude of respondents (mean PCA axis score of 6.35 [SE 0.04], equivalent to 16.15 [0.10] on the original 21‐point Likert scale) was significantly greater than the PCA score equating to neither agree nor disagree (3.9, equivalent to 10) (*t* = 64.03, df = 1165, *p* = 2.2 × 10^−16^).

**FIGURE 4:**
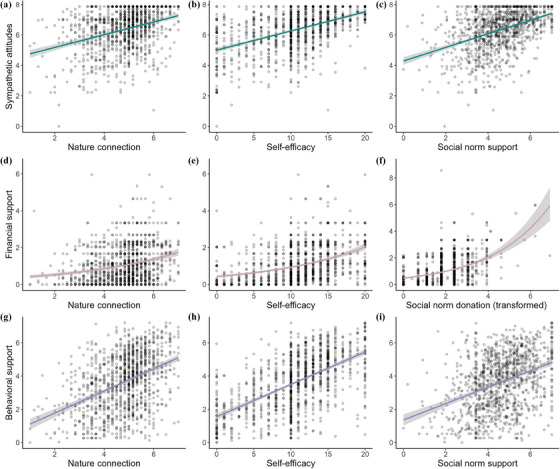
Outcome support variables used to measure support for conservation advertisements through quantitative survey questions, (a, b, c) sympathetic attitudes, (d, e, f) financial support, and (g, h, i) behavioral support relative to the predictor variables (a, d, g) nature connection, (b, e, h) self‐efficacy, and (c, f, i) perceived social norms of behavioral or financial support. All associations are statistically significant (Table 2) (gray shading, 95% confidence intervals). Sympathetic attitudes and behavioral support were fitted with a linear model, and financial support was fitted with generalized linear model with a quasi‐Poisson distribution. See the legend of Figure 2 for more information on how the response variables were calculated.

Neither message framing (Table 2; Figure 2b) nor the dynamic norm nudge significantly affected sympathetic attitudes (Table 2; Figure 3b). Psychological factors positively associated with sympathetic attitudes were nature connection (Figure 4a), self‐efficacy (Figure 4b), Global South awareness, psychological benefits, and social norm support (Figure 4c) (Table 2). Psychological factors negatively associated with sympathetic attitudes were flood experience and climate change skepticism (Table 2). Education level was negatively associated with sympathetic attitudes (Table 2), although this result became only marginally significant after FDR adjustments (Appendix S8). Older people showed less sympathetic attitudes (Table 2). Exploratory analyses revealed a significant interaction between climate change skepticism and message framing, with individuals who reported greater skepticism showing reduced sympathy toward the global ES framing (Appendix S9).

### Financial support

Respondents’ reported donations were significantly >0 (*z* = 25.6, *p* = 2.2 × 10^−16^, mean [SE] = £21.20 [1.60], median £10.00). The amount of the hypothetically available £100 for a charitable donation that respondents allocated to the campaign was also significantly >0 (*z* = 27.4, *p* = 2 × 10^−26^, mean = £40.00 [1.00], median £30.00).

Message framing did not affect financial support (Table 2; Figure 2c). Advertisements containing the dynamic norm nudge received significantly less financial support (Table 2; Figure 3c). Psychological factors that affected financial support, all positively, were nature connection (Figure 4d), self‐efficacy (Figure 4e), and social norm donation (Figure 4f). Older people showed less financial support (Table 2).

### Behavioral support

Behavioral support of respondents (mean PCA axis score of 3.63 [SE 0.05]) was not different from the PCA score equating to neither agree nor disagree on the original 21‐point Likert scale (*t* = 0.70, df = 1165, *p* = 0.484).

Message framing did not affect behavioral support (Table 2; Figure 2d). Advertisements containing the dynamic norm nudge received significantly less behavioral support than the control (Table 2; Figure 3d), although this result was marginally significant after FDR adjustments (Appendix S8). Psychological factors that positively affected behavioral support were nature connection (Figure 4g), self‐efficacy (Figure 4h), social norm support (Figure 4), and psychological benefits (Table 2). The only psychological factor that negatively affected behavioral support was climate change skepticism (Table 2). IMD was negatively associated with behavioral support, and males showed significantly less behavioral support than females or other genders (Table 2). Older people showed significantly less behavioral support (Table 2). Exploratory analyses revealed a significant interaction between psychological benefits and the norm nudge, with individuals who reported greater psychological benefits showing increased behavioral support when the nudge was present (Appendix S10).

## DISCUSSION

### Study limitations

We captured self‐reported responses to a hypothetical campaign that mimics actual interventions. A power analysis ensured the potential to detect small effects across a respondent pool representative of the UK public regarding key sociodemographic traits, although the study was underpowered to test interactions robustly. Further, some of our results and recommendations were based on observational patterns rather than on experimental tests. Reliance on a hypothetical campaign somewhat limits inference because, although outcome variables were high in internal control, they were low in external validity. Therefore, although our results provide useful insight to inform the design of actual conservation campaigns, they require real‐world validation.

We observed potential ceiling effects in sympathetic attitudes (most responses were at the higher end of the scale) and potential floor effects for financial support (most responses were at the lower end of the scale). Our survey was conducted during the COVID‐19 pandemic when some people had reduced incomes, which could have suppressed financial support relative to other years. It is thus possible that sensitivity to detect the effects of manipulations on sympathetic attitudes and financial support was reduced. We consider this unlikely, however, because we found statistical associations between these outcome variables and many of our predictors. In addition, our financial support metric was derived from 2 measures, one of which was not reliant on respondents’ personal finances, and loaded equally onto a single PCA axis. We thus consider that the pandemic's financial impacts had limited influence on our results. Rather, the contrasting ceiling (sympathetic attitudes) and floor (financial support) effects probably reflect frequent observations that pro‐environmental attitudes are stronger than pro‐environmental behaviors (Liu et al., 2020; Siegel et al., 2018).

Manipulation checks revealed that the dynamic social norm nudge treatment did not affect perceived social norms regarding donation but reduced perceived social norms for support. We were thus cautious when considering the implications of our findings for dynamic social norm theory. Despite these limitations, we consider it important to discuss the negative impacts of the dynamic social norm nudge, given that other research has indicated their potential ineffectiveness for promoting pro‐environmental behavior (Aldoh et al., 2021; Boenke et al., 2022) (see “Dynamic social norm nudges versus perceived static social norms”).

### Overall support

Our sample population exhibited strong sympathetic attitudes and financial support for tropical forest restoration. In contrast, behavioral support (sharing information, altering purchasing behavior, and providing time to support the campaign) was more limited, with participants typically being neither likely nor unlikely to exhibit such support. This finding highlights the tendency for people to express stronger pro‐environmental attitudes than actions, especially when pro‐environmental decisions generate personal costs and sacrifice (Wyss et al., 2022). Our results highlight the potential influence of moral licensing in influencing decisions, where people refrain from behavioral changes because they feel that providing financial resources eliminates the need to alter behavior (Urban et al., 2023; Xiong et al., 2023). Given the necessity of widespread behavioral shifts for global conservation goals, this poses a dilemma for ENGOs dependent on public support.

Advertisement sufficiency increased with age, suggesting younger people wanted additional information. Several mechanisms could generate this pattern, including greater trust among older individuals or higher engagement in younger people prompting them to seek additional information. The latter seems more likely given that sympathetic attitudes and behavioral and financial support were higher in younger people. This informs discussion about how pro‐environmental behavior changes with age and provides support for the socioemotional selectivity theory that, as people age, they prioritize more immediate situations over concern for the future (Carstensen et al., 2003; Wang et al., 2021). Education was negatively associated with advertisement sufficiency, suggesting educated people are more critical and thus seek additional detail. Although educational status was negatively associated with sympathetic attitudes, it was not associated with any of our support measures, further evidencing a disparity between attitudes and behaviors. Individuals from deprived neighborhoods showed higher behavioral support, potentially due to heightened awareness and support for charitable actions, although the influence of socioeconomic status on supporting charitable activity is complex and context dependent (GGSC, 2018).

### Audience segments stronger predictors of support than message framing

Our study empirically assessed the ability of conservation adverts to elicit public support across varying message framing strategies, by testing whether the addition of 2 ES framings (representing global‐scale and local‐scale benefits) to biodiversity framing increased support. Adding ES framings had no effect on any of the outcome variables compared with the biodiversity control treatment. Although a slight trend toward higher support for local‐scale ES framing was observed across all outcome variables, we found no significant difference in the ability of different ES framings to elicit support, except for the local‐scale ES framing being more effective among climate change skeptics. Our results are consistent with emerging evidence that focusing on ES, rather than the intrinsic value of nature, may not effectively strengthen public support for conservation. A recent empirical study also reported null results of message framing for conservation donations outside of a laboratory setting and across cultures (Blake et al., 2023). Other studies interpret similar findings as a negative consequence of monetized ES framings, which can neglect emotional mechanisms such as moral obligation (Batavia et al., 2018) and encourage feelings that governments or industry benefitting from investments should pay for environmental protection (Goff et al., 2017). However, our ES framing was non‐monetized, so these mechanisms were unlikely here. We suggest that message framing around ES benefits for vulnerable people may require incorporation of additional techniques, such as focusing on personal stories to enhance public resonance (Fernández‐Llamazares & Cabeza, 2018). Additional research that tests alternative approaches to ES framing is needed, alongside the assessment of respondent perceptions of the effectiveness of those framings in highlighting ES benefits. We focused on assessing the impacts of adding ES framings to conservation‐focused ENGOs’ default biodiversity messaging, but future studies could incorporate a passive control treatment (i.e., promoting no benefits to wildlife or humans) to check that benefits arise from active message framing in conservation contexts, even when no differences are found between framings (Nelson et al., 2021).

Participants who reported greater psychological benefits from charitable activities found the advertisements’ information more sufficient and exhibited greater sympathetic attitudes and behavioral support. This finding suggests that positive psychological effects of charitable activity (such as feeling good about oneself, i.e., warm glow theory [Andreoni 1990]) can drive charitable support. We suggest that incorporating statements in advertisements that emphasize the personal benefits of supporting the campaign (e.g., a person's identity as a good citizen [Ferguson et al. 2023]) will enhance psychological benefits and increase charitable support. In addition, respondents who reported higher self‐efficacy showed increased advertisement sufficiency, sympathetic attitudes, and behavioral and financial support. We suggest that incorporating impact statements within advertisements, highlighting outcomes of supporting the campaign, may secure positive responses by preventing feelings of helplessness that act as barriers to pro‐environmental behavior (Landry et al., 2018; Le et al., 2022). Statements that focus on concrete, low‐construal‐level actions (i.e., how to make a difference) are more effective than abstract, high‐construal‐level actions (i.e., why one should make a difference) because they shorten the psychological distance between the responder and intervention benefits (Grazzini et al., 2018). Impact statements might include transparent breakdowns of donation allocations and tangible outcomes (e.g., “Your donation of £X will enable us to restore X ha of essential habitat for wildlife.”).

We found that people with a stronger nature connection consistently possessed higher sympathetic attitudes and showed more behavioral and financial support. These results are consistent with the expanding amount of literature advocating for enhancing individual connection to nature to increase support for conservation, consequently removing the need for external incentives to act pro‐environmentally (Carr & Hughes, 2021; Coldwell & Evans, 2017; Richardson et al., 2020). Changing internal attitudes is extremely challenging because an individual's connection to nature is a multidimensional construct that is determined by coalescing psychological factors, including biospheric attitudes and environmental identity (Hoot & Friedman, 2011; Martin & Czellar, 2017), but a strengthened connection may arise from interventions that increase exposure to green space or nature (Coldwell & Evans, 2017; Sheffield et al., 2022).

Respondents with awareness of environmental degradation in the Global South had increased sympathetic attitudes but did not exhibit increased behavioral or financial support. These results suggest that awareness of environmental degradation in the Global South perhaps failed to shorten the psychological distance between the responder and restoration benefits (McDonald et al., 2015). Climate change skepticism was negatively associated with behavioral support regardless of message framing, although effects were slightly reduced for local ES framings—this was unsurprising given their limited focus on carbon storage effects. This is consistent with findings that personal knowledge relating to a conservation message influences engagement (Reichl et al., 2021; Scannell & Gifford, 2013). Flood experience did not increase support for campaigns; however, this could relate to the low average flood experience among respondents (Appendix S6). Personal experience with flooding also had no influence on behaviors that reduced personal carbon footprint (Lohmann & Kontoleon, 2023). The negative relationship between flooding and sympathetic attitudes could be attributed to feelings of fear induced by the perceived increased proximity of a threat, which subsequently prevents engagement with information highlighting those threats (Brügger et al., 2016; Hart et al., 2015).

### Dynamic social norm nudges versus perceived static social norms

We assessed the effectiveness of a descriptive dynamic social norm as a nudge to increase support for conservation advertisements. Contrary to our hypothesis, the addition of dynamic social norms to advertisements decreased perceived sufficiency and financial and behavioral support. Descriptive norms can backfire by inducing behavioral shifts that are opposite to those intended, generating boomerang effects (Ozaki & Nakayachi, 2020; Schultz et al., 2007). Boomerang effects can occur when individuals perceive the norm as overly controlling, prompting deviant behavior, which our phrase “Will you join them?” may have encouraged. However, this phrase could also imply some societal judgment of people who do not exhibit such support, resulting in our phrase including elements of an injunctive norm (i.e., emphasizing expectations of others’ approval or disapproval), which can eliminate boomerang effects (Schultz et al., 2007). Studies reporting positive effects of dynamic norms tend to use descriptive norms (e.g., Sparkman & Walton 2017). However, recent work suggests descriptive and injunctive norms are equally but uniquely effective at shifting behavior and could be utilized in combination (Farrow et al., 2017; Helferich et al., 2023). Therefore, the context or culture in which the norm is applied, rather than the norm type, is likely responsible for an individual's desire to conform. For example, although the provision of dynamic norms to influence meat consumption was effective in the United States (Sparkman & Walton, 2017), the use of an equivalent norm in the United Kingdom (our study location) was ineffective (Aldoh et al., 2021). Moreover, the effectiveness of dynamic norms can be influenced by the messenger's perceived trustworthiness, with messages delivered by activists being ineffective even when the same message was delivered effectively by a researcher (Boenke et al., 2022). Conservation marketing messages, such as our experiment, could seem activist driven, potentially weakening their effectiveness and posing challenges for implementing dynamic norms in conservation.

The negative impact of the nudge on perceived social norm support could arise because dynamic norms typically emphasize that focal behaviors are not yet mainstreamed in the population. Dynamic norms can fail to increase respondents’ perceptions of the frequency of focal behaviors exhibited by the public (Dimant et al., 2020), likely leading to behaviors being perceived as less socially acceptable and less desirable to adopt. Social identity theory suggests dynamic norm boomerang effects are particularly likely when respondents are influenced by peers not conforming to the advertised norm (Ozaki & Nakayachi, 2020).

In contrast, we found positive effects of static social norms, where perceived support from others increased respondents’ support. Therefore, the free‐rider issue (sensu Bicchieri & Dimant 2019) (i.e., people not providing support because they believe others will) was not prevalent among our study population. This highlights the importance of understanding individual beliefs, barriers, and contexts to changing behavior (Boenke et al., 2022; Çoker et al., 2022; Hauser et al., 2018). We interpreted the contrasting effect of dynamic social norm nudges and existing static social norms as evidence that our dynamic norm nudge was incapable of shifting the perception of existing norms (Blumenthal et al., 2001). This is a fundamental risk of dynamic norms because, by design, they focus on temporal changes in attitudes rather than describing the typical behavior. It remains plausible that dynamic norms could effectively enhance conservation marketing targeted at individuals more inclined to be trend setters rather than trend followers. More evidence is required to assess the effectiveness of norm nudges within a conservation marketing context, especially how dynamic norms interact with static norms. Regardless, our work highlights that dynamic norm nudges can adversely affect public support for conservation, and contextual factors should be carefully considered if incorporating such nudges into marketing designs.

### Recommendations

Our study makes 2 important theoretical contributions. We highlighted situations where dynamic social norms can backfire and identified potential moral licensing issues, where seeking financial support in conservation advertisements could reduce the uptake of the behavioral changes required to meet global conservation targets. We also provided practical insights that can inform strategies to increase public support for conservation. First, although ES message framings work in policy contexts, adding such framings to biodiversity‐focused messages is unlikely to be effective. Second, we caution against the use of dynamic social norm nudges in a conservation context without careful consideration of relevant audience segments. Third, including impact statements highlighting specific outcomes from an individual's support may increase engagement by enhancing self‐efficacy and reducing psychological distance. Finally, strategies that increase nature connectedness are essential to strengthen public support for conservation.

## AUTHOR CONTRIBUTIONS

Dina‐Leigh Simons and Karl Evans conceived the study and led its design with assistance from Richard Bradbury. Data collection and statistical analyses were conducted by Dina‐Leigh Simons with assistance from Karl Evans. Dina‐Leigh Simons and Karl Evans led the interpretation of the results. Dina‐Leigh Simons led the writing of the manuscript with assistance and feedback from Karl Evans and Richard Bradbury. Karl Evans was the supervisor of Dina‐Leigh Simons throughout the project.

## Supporting information



Supplementary Materials
