# Supplementary material for: Impacts of ecosystem service message framing and dynamic social norms on public support for tropical forest restoration
Source: Conserv Biol. 2024 Sep 10;39(2):e14373. doi: 10.1111/cobi.14373 (PMC11959341; doi:10.1111/cobi.14373)
Supplement: Supplementary file 1 — Supplementary Materials [file COBI-39-e14373-s001.docx]

# Supporting information

Additional supporting information may be found in the online version of the article at the publisher’s website.

**Appendix S1:** Pilot study and respondent recruitment

The study was piloted with 20 people to resolve issues regarding clarity and user experience (17^th^-23^rd^ March 2020) and then refined before it was released onto Prolific (22^nd^-27^th^ April 2020). The survey title and description did not reference any conservation activities, to prevent bias towards respondents who were interested in such topics (Appendix S3). When opening the questionnaire, further instructions on how to complete the survey and an ethics statement were revealed (Appendix S4).

**Appendix S2:** Deriving socio-demographics

Financial security was recorded by four quantitatively measured questions adapted from Goff et al (2017): i) *‘I usually have enough money to go on holiday abroad’,* ii) *‘I often worry about being able to pay my monthly utility bills, such as heat, water, or electricity’* (reverse coded), iii)*‘I am often able to purchase luxury items, such as jewellery or designer clothing’* and iv) *‘Being able to pay my rent or mortgage payments is a constant concern*’ (reverse coded). Following the standard approach for using this scale, values were averaged to create a single continuous variable. Age was treated as a continuous variable by taking the mid-point of each of eight adult age ranges (18-24, 25-34, 35-44, 45-54, 55-64, 65-74, 75-84, 85-94). Gender was measured as a 3-level categorical variable (male, female, other). For IMD, deciles were extracted from the latest national IMD online postcode identification tools, where one equates to most deprived neighbourhoods and ten equates to least deprived neighbourhoods (Office for National Statistics, 2019). Education was treated as a continuous variable by assigning numeric values to the five categories measured (none - 1, O level/GCSE or equivalent including vocational equivalents - 2, A level or equivalent including vocational equivalents - 3, Undergraduate degree - 4, higher degree - 5). Ethnicity was recorded by 5-level categorical factor (White or Caucasian; Black/African/Caribbean, Black British; Asian or Asian British; Mixed/Multiple ethnic groups; other).

**Appendix S3:** Survey title and information shown on Prolific before respondents accepted to take part in the experiment.

**‘Assessing responses to charity campaign adverts.’**

We are interested in understanding how people respond to charity adverts and why for a research project. In this study, you will be asked to look at a mock charity advert and complete a questionnaire. This will take between 10-15 minutes. The questionnaire will record your opinions about the advert and related issues as well as asking for a small amount of general information about you, such as if you can afford to buy luxury items. You will need to complete the questionnaire in one sitting with minimal breaks or interruptions. It is important that you answer every question in the questionnaire otherwise your submission may be rejected, and you won’t get rewarded. You can use the ‘exit’ button within the study at any time if you no longer want to participate.

**Appendix S4:** Full questionnaire released on Survey Monkey (instructions and Sections 1-11). Each section of the questionnaire was shown on a new online page.

***Questionnaire instructions***

We are interested in understanding how people respond to charity adverts and why for a research project. This questionnaire will record your opinions about a charity advertisement as well as asking for a small amount of general information about yourself, such as if you can afford to buy luxury items. There are no right or wrong answers.

**There are three different types of questions that require different actions from you.**

1.     **Slider bar questions** – Slide the circle to the place along the bar that you feel best represents your response. You can place the slider anywhere along the bar. The box next to the slider shows your answer. You must click or move the slider for your response to be recorded – even if you want to return it to its original position. 
2.     **Tick box questions** - select the box which best describes your response.
3.     **Open questions** - write your answer in the text boxes. 
 
Please read all of the instructions carefully and complete the questionnaire in one sitting with minimal breaks or interruptions. It is important that you answer every question in the questionnaire, otherwise we may have to reject your submission and you will not be rewarded.
 
This questionnaire has 11 sections. Each section will take between 1 and 2 minutes to complete. Sections 2-7 refer specifically to the charity campaign detailed in the advert. Sections 1, 8-11 refer to your own opinions and how you feel towards specific statements.
 
If at any point you no longer wish to participate, you can use the ‘exit’ button within the study. The Prolific completion URL and completion code will be revealed to you after you have submitted your response. Please open the URL and enter the code to prove that you have completed the study.

According to data protection legislation, we are required to inform you that the legal basis we are applying in order to process your personal data is that ‘processing is necessary for the performance of a task carried out in the public interest’ (Article 6(1)(e)). Further information can be found in the University’s Privacy Notice *web address blinded for double blind review*. As we will be collecting some data that is defined in the legislation as more sensitive (information about personal finance and residence), we also need to let you know that we are applying the following condition in law: that the use of your data is ‘necessary for scientific or historical research purposes’.
 
Contacts:
1)    *name blinded for double blind review* – *address blinded for double blind review* (*e-mail blinded for double blind review*)
2)    *name blinded for double blind review* - *address blinded for double blind review* (*e-mail blinded for double blind review*)

Please enter your Prolific ID:

[text box – open ended]

**If you are completing this survey on a mobile device or tablet, please hold it in portrait view.**

**
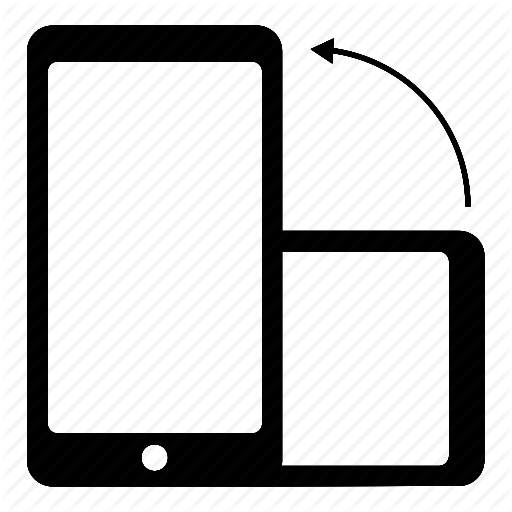
**

***Section 1***

(1a) If you were given £300 to give to charities from the following sectors, how many pounds (£, if anything) would you give to each type of charity? Please allocate all the £300 to only the three groups below. *Insert the numeric value only – please don’t insert £ before your answer.*

Wildlife conservation and protecting the environment [free text box]

Supporting vulnerable people in UK [free text box]

Supporting vulnerable people in developing countries [free text box]

***Section 2***

[advert presented here (determined by randomisation tool)]

(2a) Which letter do you see in the top left-hand corner of this advert?


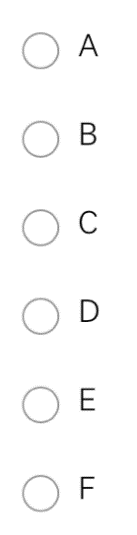


***Section 3***

***Please fully read the following advertisement in detail before answering any questions. You can refer back to the advert at any point by scrolling to the top of the page.***

[advert presented here]

***On a scale from 0 (strongly disagree) to 20 (strongly agree), how much do you agree with the following statements?***

(3a) I felt supportive towards this advert.


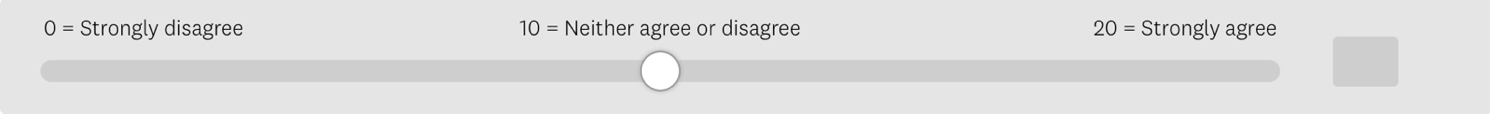


(3b) I hope that this campaign for rainforest restoration succeeds.


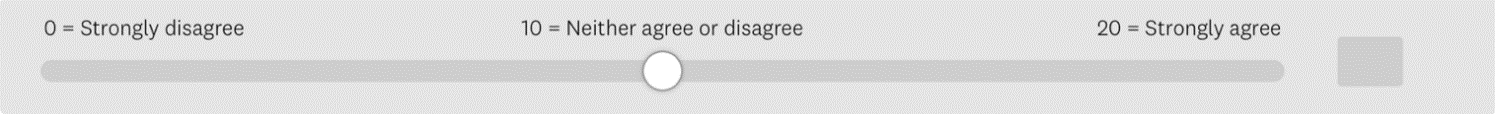


***Section 4***

[advert presented here]

(4a) How many pounds (£) would you be willing to give as a one-off donation to this campaign? If you would not donate, put 0. *Insert the numeric value only – please don’t insert £ before your answer.*

[free text box]

(4b) What are the main reasons why you did, or did not, donate?

[free text box]

(4c) If you were given £100 to support any charitable activity, how much would you allocate to support the advert's campaign? If you would not donate, put 0. *Insert the numeric value only – please don’t insert £ before your answer.*

[free text box]

***Section 5***

[advert presented here]

***On a scale from 0 (extremely unlikely) to 20 (extremely likely), please answer the following questions.***

(5a) How likely are you to sign a petition (either online or on paper) to restore African rainforest as mentioned in the advert?


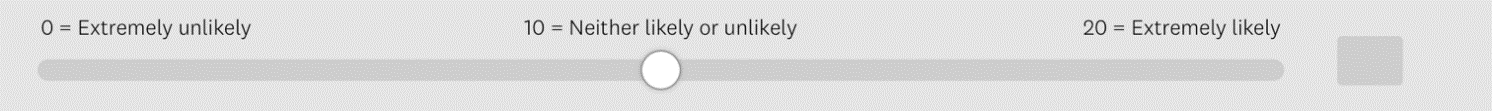


(5b) Over the next week, how likely are you to talk about this campaign in a conversation with someone to help it succeed?


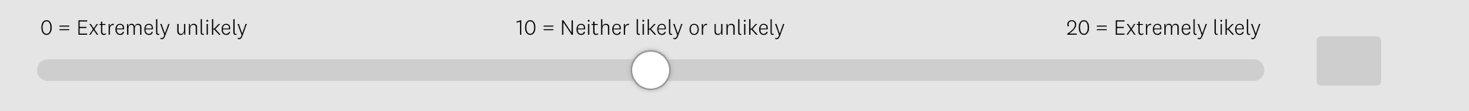


(5c) If this advert was posted on one of your social media platforms, how likely are you to share it on that platform to help it succeed (e.g. using the “share” button on Facebook, the “retweet” button on Twitter, using the “repost to story” button on Instagram etc.)?


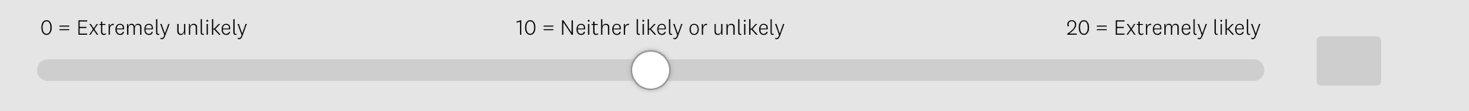


(5d) How likely are you to buy furniture or another wood product that is exactly what you are looking for if you found out they were made using wood from this east African rainforest and hindered forest recovery?


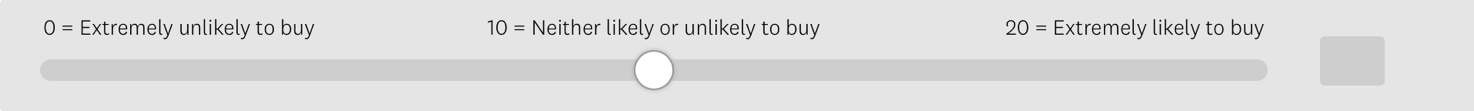


(5e) If given the opportunity, I would give up my time to volunteer at a local charity event in my own town to help raise funds to support this rainforest restoration campaign.


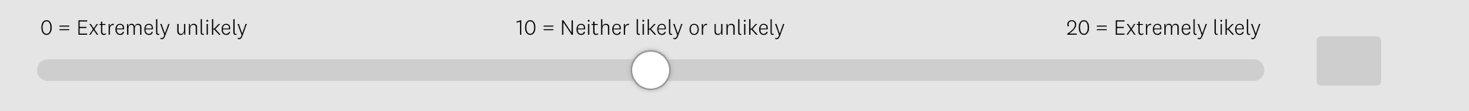


***Section 6***

[advert presented here]

***On a scale from 0 (strongly disagree) to 20 (strongly agree), how much do you agree with the following statement?***

(6a) The advert alone gave me enough information to decide whether to support this campaign.


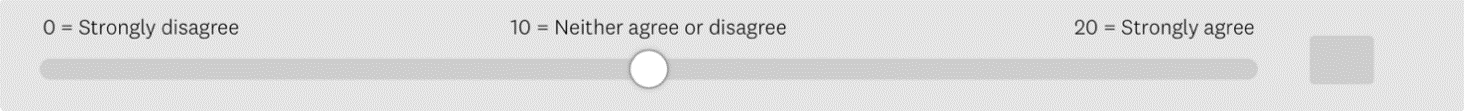


(6b) Did you use your existing knowledge to help you decide whether or not to support the campaign? If so, then what knowledge/facts did you use? If you only used information in the advert, then write "none" in the box.

[free text box]

(6c) Was there any additional information not included in the advert that you would have liked to know to help you decide whether to support the campaign or not? If so, what information would you have liked? Otherwise write "none" in the box.

[free text box]

***Section 7***

[advert presented here]

***On a scale from 0 (strongly disagree) to 20 (strongly agree), how much do you agree with the following statements?***

(7a) Most other members of the public would hope that this campaign succeeds.


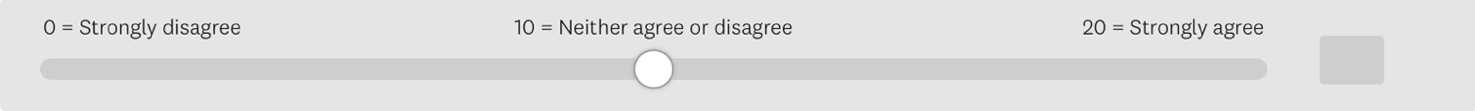


(7b) Most other members of the public would support this campaign.


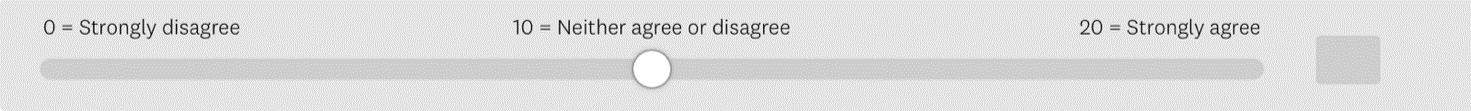


(7c) On average, how many pounds (£) do you think other people would donate as a one-off-donation to this campaign? *Insert the numeric value only – please don’t insert £ before your answer.*

[free text box]

(7d) I believe that if I supported this campaign it would make an impactful difference.


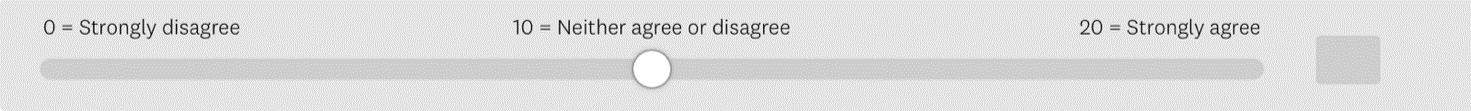


(7e) Supporting a charitable cause makes me feel better in myself.


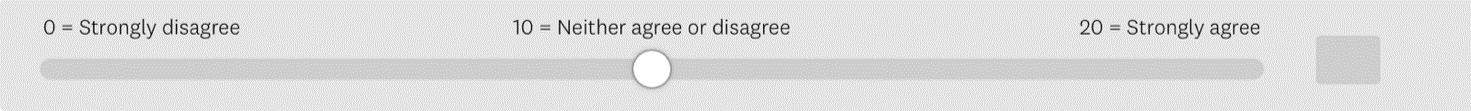


(7f) I often feel regret or guilt if I don’t support a charitable cause after being asked to.


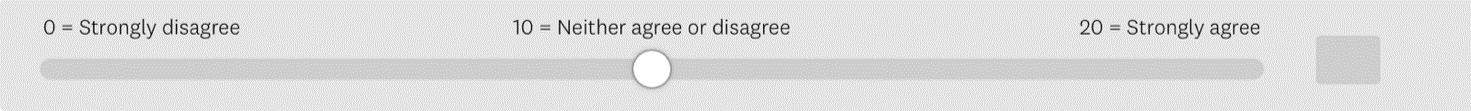


***Section 8***

***On a scale from 0 (strongly disagree) to 20 (strongly agree), how much do you agree with the following statements?***

(8a) I usually have enough money to go on holiday abroad.


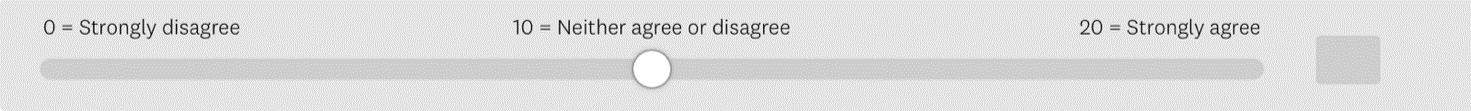


(8b) I often worry about being able to pay my monthly utility bills, such as heat, water, or electricity.


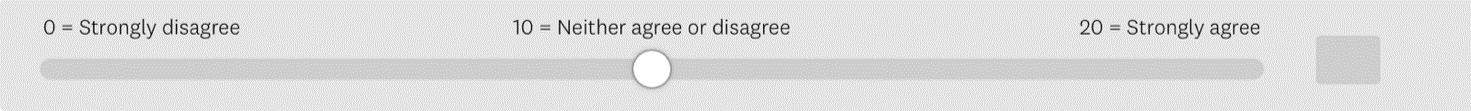


(8c) I am often able to purchase luxury items, such as jewellery or designer clothing.


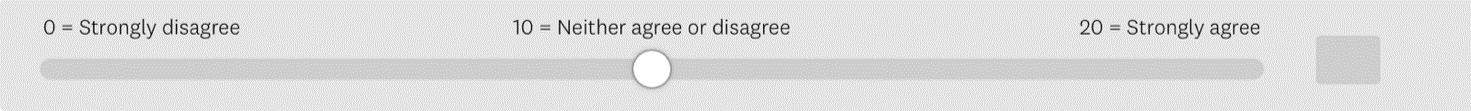


(8d) Being able to pay my rent or mortgage payments is a constant concern.


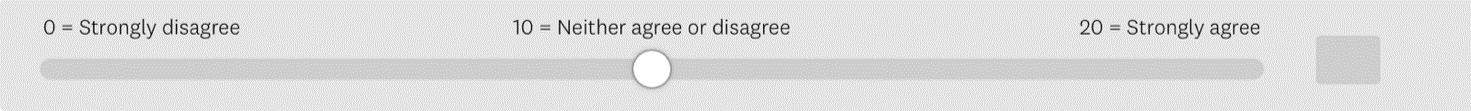


***Section 9***

***On a scale from 0 (strongly disagree) to 20 (strongly agree), how much do you agree with the following statements?***

(9a) I have personally witnessed **in real life** the adversity people face in poor, developing countries.


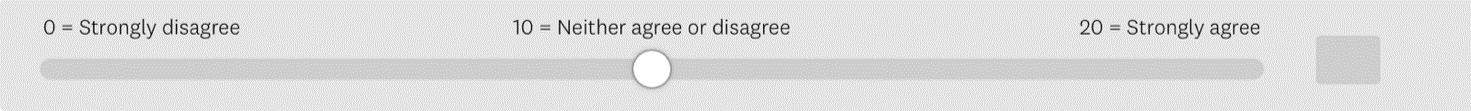


(9b) I have personally witnessed **through television, online or local campaigns,** the adversity people face in poor, developing countries.


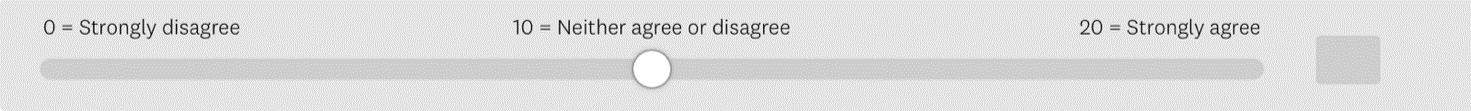


(9c) I believe people are suffering in Africa because of environmental degradation caused by human activities.


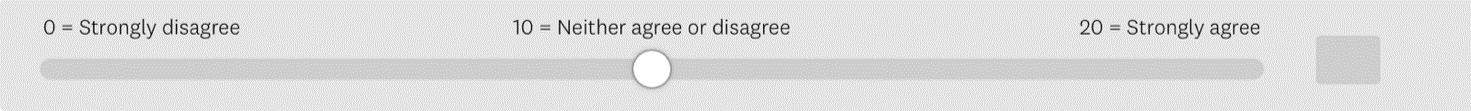


(9d) Claims that human activities are changing the climate are exaggerated.


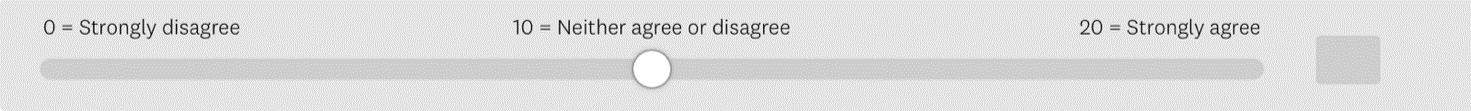


(9e) Climate change is just a natural fluctuation in the Earth’s temperatures.


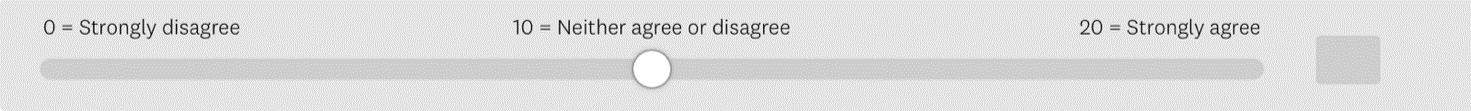


(9f) I believe climate change is a real problem.


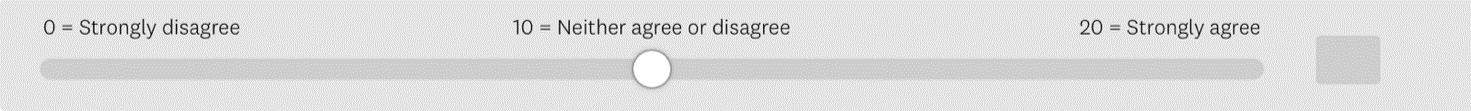


(9g) How many flood events have influenced your life in the following ways over the last five years (i.e. between this date in 2015 and today)?


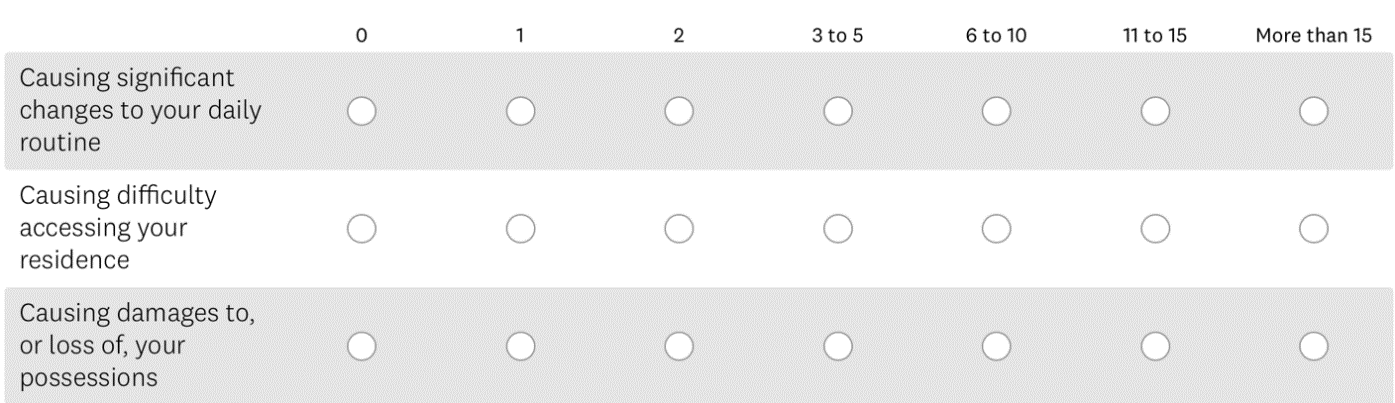


***Section 10***

***For each of the following, please rate the extent to which you agree with each statement, using the scale from 1 to 5 as shown below. Please respond as you really feel, rather than how you think “most people” feel.***

(10a) My ideal vacation spot would be a remote, wilderness area.


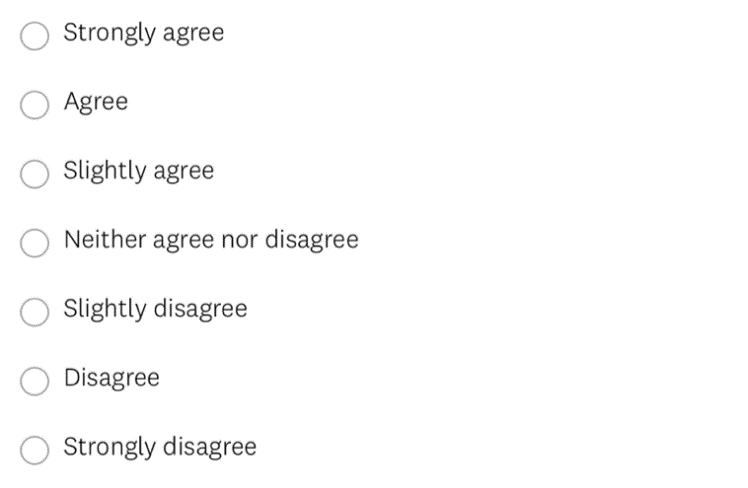


(10b) I always think about how my actions affect the environment.


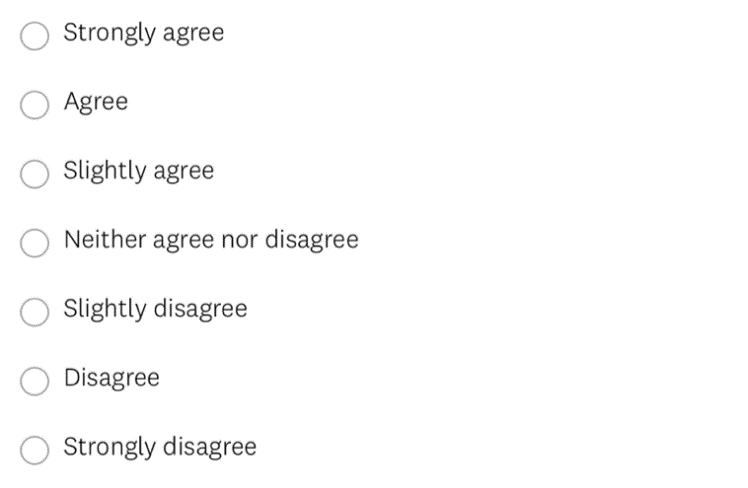


(10c) My connection to nature and the environment is a part of my spirituality.


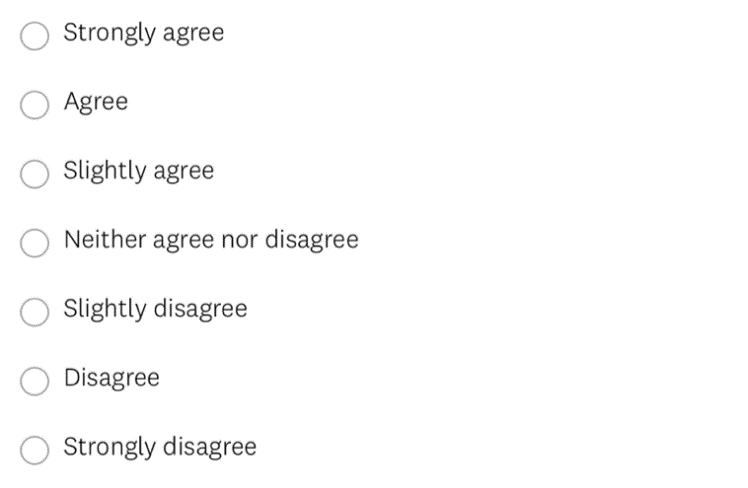


(10d) I take notice of wildlife wherever I am.


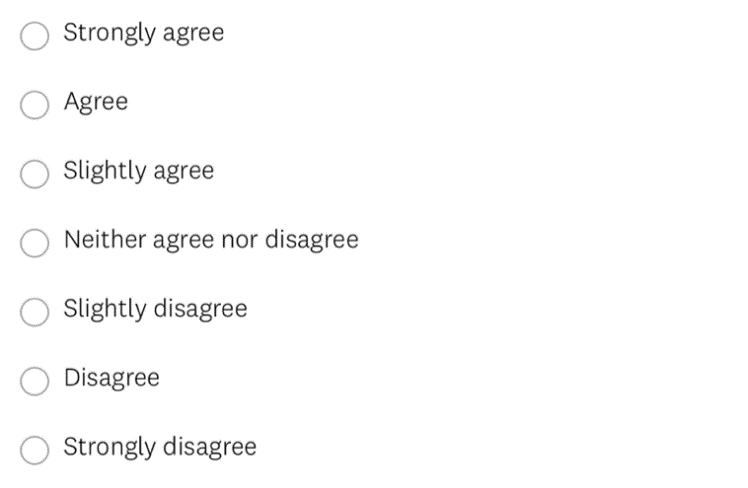


(10e) My relationship to nature is an important part of who I am.


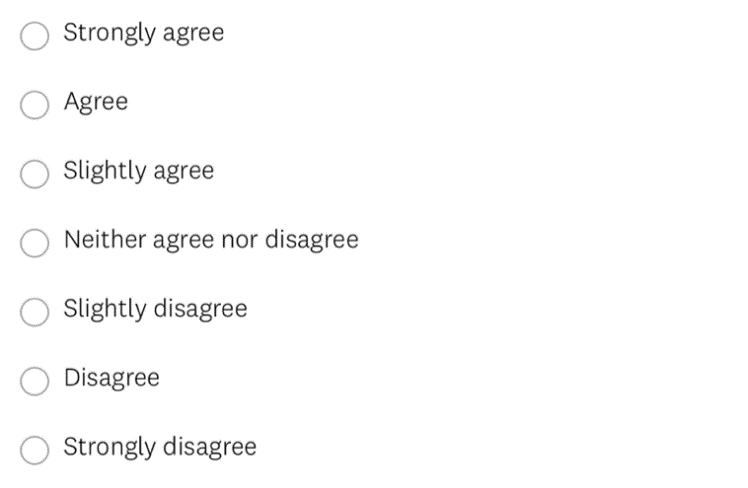


(10f) I feel very connected to all living things and the earth.


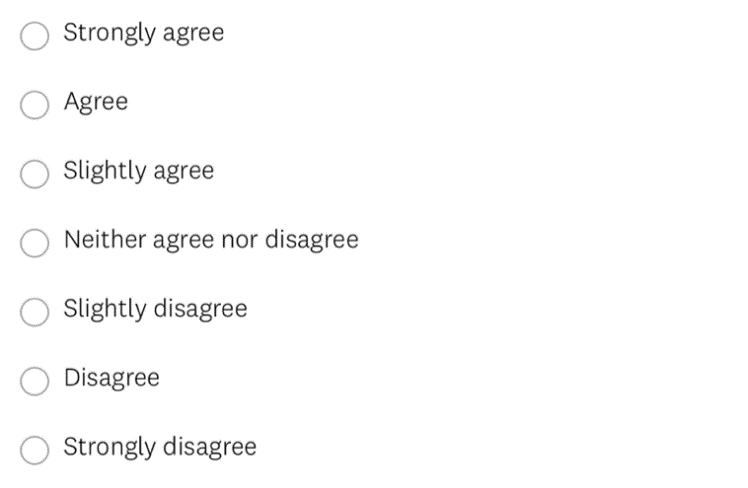


***Section 11***

(11a) What gender are you?


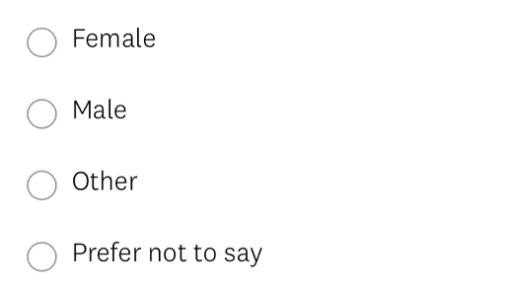


(11b) What is your age?


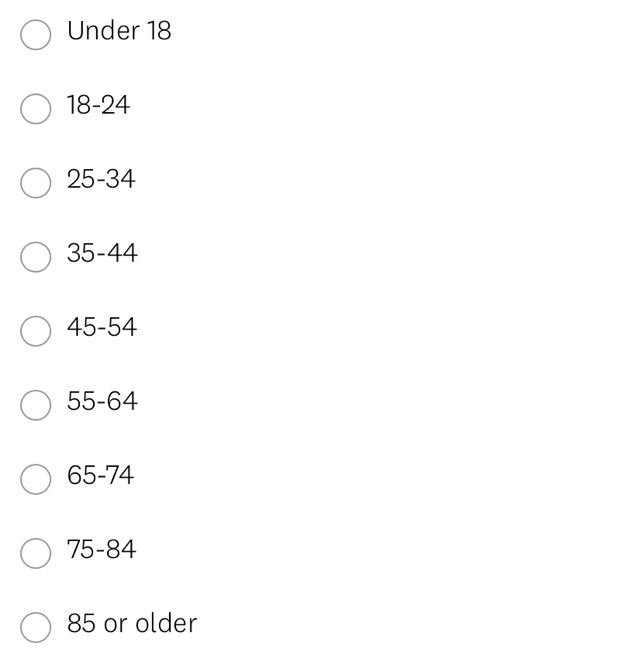

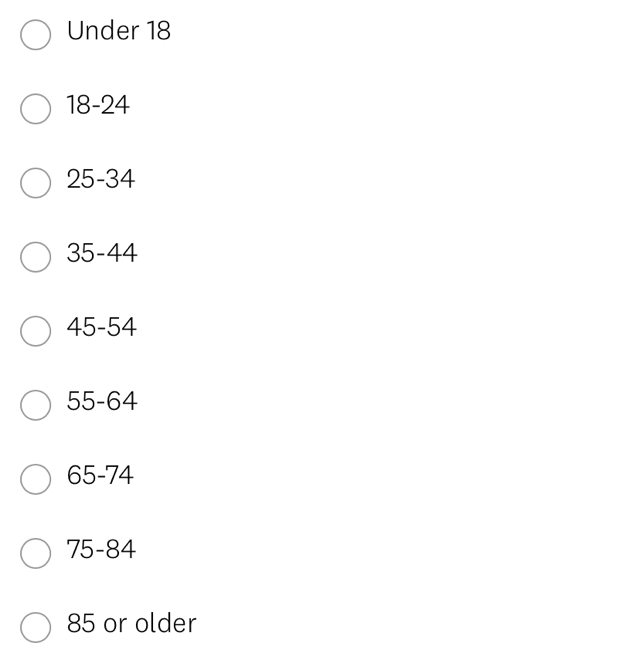


(11c) What is your ethnicity?


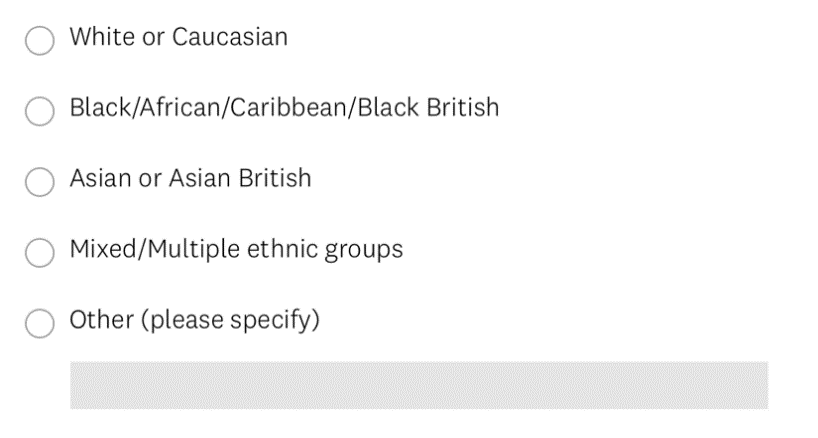


(11d) What is the highest level of qualification you have?


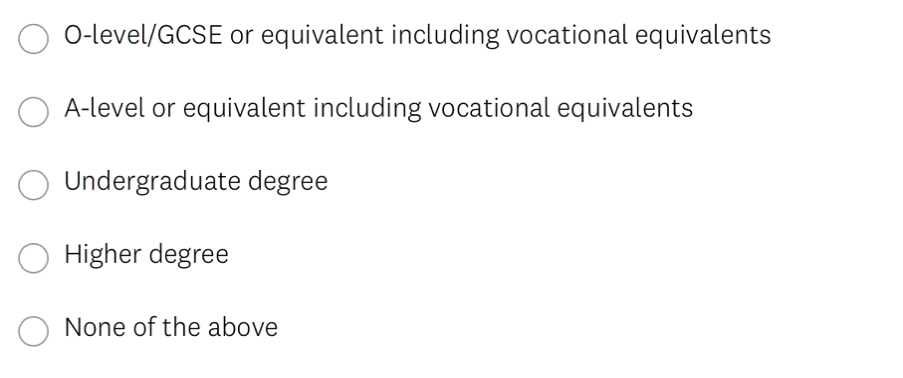


(11e) What is your full postcode (e.g. S6 2WF or SG19 1AD)?

[free text box]

**Press Done to submit your response**

After submitting your response, the Prolific completion URL and completion code will be revealed to you on the next page. Please follow the instructions on the next page to finish the survey and ensure that you are rewarded by Prolific. 

Thank you for participating.

**Appendix S5:** Socio-demographic profile of respondents recruited by Prolific (*n=*1166) in comparison to the 2011 UK Census data for each treatment. Shaded columns show profiles as the percentage (%) of the sample for each level of the demographic trait for the stated treatment. UK census age data are rescaled across the adult age groups to aid comparison.

| **Demographic** | **Category** | **UK Census** | **Total Study** | **BD** | **BD+ES-L** | **BD-ES-G** | **Nudge present** | **Nudge absent** |
| --- | --- | --- | --- | --- | --- | --- | --- | --- |
| Age | 18 to 24 | 11.9 | 10.5 | 10.6 | 10.0 | 10.8 | 10.4 | 10.5 |
|  | 25 to 34 | 17.0 | 18.8 | 18.3 | 20.1 | 18.1 | 21.1 | 16.4 |
|  | 35 to 44 | 17.8 | 18.0 | 18.5 | 17.2 | 18.3 | 18.4 | 17.6 |
|  | 45 to 54 | 17.4 | 16.4 | 18.8 | 13.5 | 16.7 | 15.3 | 17.4 |
|  | 55 to 64 | 14.9 | 25.0 | 24.8 | 28.0 | 22.4 | 23.7 | 26.4 |
|  | 65 to 74 | 11.1 | 10.2 | 7.9 | 10.8 | 12.1 | 9.9 | 10.5 |
|  | 75 to 84 | 7.1 | 1.0 | 1.2 | 0.3 | 1.6 | 1.2 | 0.9 |
|  | 85 to 94 | 2.8 | 0.1 | 0.0 | 0.3 | 0.0 | 0.0 | 0.2 |
| Gender | Female | 51.0 | 50.8 | 48.6 | 50.1 | 53.9 | 48.6 | 53.0 |
|  | Male | 49.0 | 48.8 | 51.0 | 49.1 | 46.1 | 51.3 | 46.3 |
|  | Other | 0.0 | 0.4 | 0.5 | 0.8 | 0.0 | 0.2 | 0.7 |
| Ethnicity | White | 86.0 | 85.2 | 87.0 | 82.8 | 85.7 | 86.9 | 83.6 |
|  | Asian | 7.5 | 7.9 | 7.2 | 8.7 | 7.8 | 7.8 | 7.9 |
|  | Black | 3.3 | 3.4 | 2.6 | 4.0 | 3.8 | 2.7 | 4.1 |
|  | Mixed | 2.2 | 1.3 | 1.9 | 2.6 | 1.9 | 1.9 | 2.4 |
|  | Other | 1.0 | 2.1 | 1.2 | 1.8 | 0.8 | 0.7 | 1.9 |
| UK nation | England | 84.3 | 86.0 | 84.9 | 85.2 | 88.1 | 84.8 | 87.2 |
|  | Wales | 4.7 | 4.4 | 3.1 | 5.8 | 4.3 | 4.8 | 4.0 |
|  | Scotland | 8.2 | 7.9 | 10.1 | 7.1 | 6.2 | 9.2 | 6.6 |
|  | Northern Ireland | 2.8 | 1.7 | 1.9 | 1.8 | 1.3 | 1.2 | 2.2 |

**Appendix S6:** Attribute profile of respondents (mean ± standard error) from raw data in the entire study, and within each treatment.

| **Demographic** | **Range** | **Total Study** | **BD** | **BD+ES-L** | **BD-ES-G** | **Dynamic norm-nudge present** | **Dynamic norm-nudge absent** |
| --- | --- | --- | --- | --- | --- | --- | --- |
| (a) Self-efficacy | 0 to 20 | 10.70 ± 0.16 | 10.48 ± 0.25 | 10.98 ± 0.25 | 10.68 ± 0.26 | 10.41 ± 0.20 | 11.00 ± 0.21 |
| (b) Nature connection | 1 to 7 | 4.80 ± 0.03 | 4.78 ± 0.05 | 4.84 ± 0.05 | 4.77 ± 0.05 | 4.83 ± 0.04 | 4.76 ± 0.05 |
| (c) Global south awareness | 0 to 7.28 | 5.01 ± 0.04 | 4.93 ± 0.06 | 5.08 ± 0.06 | 5.03 ± 0.06 | 5.01 ± 0.05 | 5.01 ± 0.05 |
| (d) Social norm ‘support’ | 0 to 7.02 | 4.62 ± 0.04 | 4.59 ± 0.06 | 4.67 ± 0.06 | 4.60 ± 0.07 | 4.48 ± 0.05 | 4.76 ± 0.05 |
| (e) Social norm ‘donation’ (£) | 0 to 5000 | 17.17 ± 4.45 | 16.26 ± 3.27 | 24.65 ± 13.19 | 10.54 ± 0.73 | 13.39 ± 2.06 | 20.99 ± 8.71 |
| (f) Climate change scepticism | 0 to 7.30 | 1.63 ± 0.05 | 1.61 ± 0.08 | 1.69 ± 0.08 | 1.61 ± 0.08 | 1.60 ± 0.06 | 1.67 ± 0.07 |
| (g) Flood experience | 0 to 11.33 | 0.35 ± 0.03 | 0.35 ± 0.05 | 0.37 ± 0.05 | 0.34 ± 0.05 | 0.40 ± 0.05 | 0.31 ± 0.04 |
| (h) IMD | 1 to 10 | 5.60 ± 0.08 | 5.78 ± 0.14 | 5.49 ± 0.15 | 5.53 ± 0.14 | 5.62 ± 0.12 | 5.59 ± 0.11 |
| (i) Financial security | 1 to 20 | 10.33 ± 0.14 | 10.30 ± 0.25 | 10.21 ± 0.25 | 10.49 ± 0.25 | 10.25 ± 0.20 | 10.41 ± 0.21 |
| (j) Education | 1 to 5 | 3.58 ± 0.03 | 3.59 ± 0.05 | 3.65 ± 0.05 | 3.49 ± 0.05 | 3.61 ± 0.04 | 3.54 ± 0.04 |
| (k) Age | 0 to 89.5 | 45.85 ± 0.46 | 45.40 ± 0.74 | 46.06 ± 0.80 | 46.13 ± 0.83 | 45.18 ± 0.64 | 46.53 ± 0.64 |

**Appendix S7:** Multiple regression models of perceived social norm ‘support’, social norm ‘donation’ and self-efficacy as manipulation checks for the norm-nudge treatment, showing parameter estimates, standard error (in parentheses) and *p*-values. *, **, *** indicates significance respectively at the α < 0.05, < 0.01, and < 0.001 level.

| **Predictor** |  | **Social norm ‘support’** | **Social norm ‘donation’** | **Self-efficacy** |
| --- | --- | --- | --- | --- |
| Dynamic norm-nudge (relative to absent) | Present | -0.274 [-0.415, -0.132]  ***p* = 2e-16******* | -0.058 [-0.167, 0.050]  *p* = 0.293 | -0.521 [-1.087, 0.046]  *p* = 0.072 |
| IMD |  | 0.028 [-0.005, 0.047]  *p* = 0.117 | 0.006 [-0.014, 0.026]  *p* = 0.544 | 0.072 [-0.032, 0.175]  *p* = 0.176 |
| Financial security |  | -0.004 [-0.018, 0.011]  *p* = 0.624 | -0.009 [-0.020, 0.003]  *p* = 0.118 | -0.039 [-0.099, 0.019]  *p* = 0.186 |
| Education |  | -0.150 [-0.221, -0.074]  ***p* = 8.9e-5***** | -0.041 [0.099, 0.015]  *p* = 0.153 | -0.309 [-0.605, -0.013]  ***p* = 0.041***** |
| Gender (relative to female) | Male | -0.130 [-0.270, 0.013]  *p* = 0.074 | -0.281 [-0.389, -0.172]  ***p* = 4.4e-7***** | -1.020 [-1.585, -0.453]  ***p* = 0.0004***** |
|  | Other | -0.184 [-0.902, 1.271]  *p* = 0.740 | 0.353 [-1.186, 0.482]  *p* = 0.407 | 0.615 [-3.737, 4.967]  *p* = 0.782 |
| Age |  | -0.012 [-0.017, -0.008]  ***p* = 3e-7***** | -0.009 [-0.013, -0.006]  ***p* = 7.7e-7***** | -0.032 [-0.051, -0.013]  ***p* = 0.001**** |
| Ethnicity (relative to white) | Other | 0.364 [0.158, 0.570]  ***p* = 0.0006***** | 0.353 [0.194, 0.511]  ***p* = 1.4e-5***** | 1.330 [0.503, 2.156]  ***p* = 0.002***** |
|  |  |  |  |  |
| **R^2 /^D^2^** |  | **R^2^ = 0.06** | **R^2^ = 0.07** | **R^2^ = 0.04** |
| **df** |  | 1157 | 1157 | 1157 |
| **F** |  | **9.20***** | **10.55***** | **5.72***** |

**Appendix S8:** Comparison between unadjusted p-values (*p*) and FDR adjusted p-values (*p_FDR_*) for main models of the four outcome variables: advert sufficiency, sympathetic values, financial support, and behavioural support. *, **, *** indicates significance levels respectively at the α < 0.05, < 0.01, and < 0.001 level. Orange highlights indicate where covariates have changed to borderline significance.

| **Predictor** | **Advert sufficiency** | | **Sympathetic attitudes** | | **Financial support** | | **Behavioural support** | |
| --- | --- | --- | --- | --- | --- | --- | --- | --- |
|  | *p* | *p_FDR_* | *p* | *p_FDR_* | *p* | *p_FDR_* | *p* | *p_FDR_* |
| Message framing | 0.098 | 0.195 | 0.351 | 0.438 | 0.114 | 0.956 | 0.963 | 0.963 |
| Dynamic norm-nudge | **0.017*** | **0.047*** | 0.781 | 0.781 | **0.006**** | **0.018*** | **0.039*** | 0.065 |
| Self-efficacy | **2.2e-16***** | **2.2e-16***** | **1.3e-13***** | **6.7e-13***** | **2.2e-16***** | **2.2e-16***** | **2.2e-16***** | **2.2e-16***** |
| Nature connection | 0.464 | 0.593 | **8.6e-12***** | **3.3e-11***** | **1.8e-9***** | **5.3e-9***** | **2.2e-16***** | **2.2e-16***** |
| Psychological benefits | **3.9e-5***** | **0.0002***** | **0.002**** | **0.006**** | 0.835 | 0.956 | **2.5e-7***** | **1.3e-6***** |
| Awareness of global South | 0.974 | 0.974 | **0.003**** | **0.008**** | 0.116 | 0.220 | 0.110 | 0.155 |
| Social norm ‘support’ | n/a | n/a | **2.2e-16***** | **2.2e-16***** | n/a | n/a | **1.8e-5***** | **0.0001***** |
| Social norm ‘donation’ | n/a | n/a | n/a | n/a | **2.2e-16***** | **2.2e-16***** | n/a | n/a |
| Climate change scepticism | 0.078 | 0.1810 | **2e-16***** | **2.2e-16***** | 0.120 | 0.220 | **0.0005***** | **0.002**** |
| Flood experience | 0.844 | 0.909 | **0.021*** | **0.039*** | 0.315 | 0.474 | 0.114 | 0.155 |
| IMD | 0.322 | 0.512 | 0.085 | 0.117 | 0.316 | 0.474 | **0.021*** | **0.039*** |
| Financial security | 0.731 | 0.853 | 0.710 | 0.768 | 0.875 | 0.956 | 0.235 | 0.252 |
| Education | **3.4e-6***** | **2.8e-5***** | **0.045*** | 0.075 | 0.713 | 0.956 | 0.169 | 0.211 |
| Gender | 0.466 | 0.593 | 0.716 | 0.768 | 0.968 | 0.968 | **0.008**** | **0.017*** |
| Age | **6e-5***** | **0.0002***** | **0.005**** | **0.01**** | **7.3e-6***** | **2.7e-5***** | **0.002**** | **0.005**** |
| Ethnicity | 0.329 | 0.512 | 0.067 | 0.1007 | 0.061 | 0.146 | 0.230 | 0.252 |

**Appendix S9:** Parameter estimates with standard errors (in parentheses) and *p*-values for interactions tests between message framing and audience segmentation variables across the four outcome variables: advert sufficiency, sympathetic values, financial support, and behavioural support. Bold indicates significance. FDR adjusted p-values (*p_FDR_*) given for significant interactions.

| **Interaction (with message framing)** | **Advert sufficiency** | | **Sympathetic attitudes** | | | **Financial support** | | **Behavioural support** | |
| --- | --- | --- | --- | --- | --- | --- | --- | --- | --- |
|  | ES-local | ES-global | ES-local | ES-global | ES-local | | ES-global | ES-local | ES-global |
| Dynamic norm-nudge | 0.620 (0.708)  *p* = 0.382 | 0.912 (0.714)  *p* = 0.202 | -0.188 (0.140)  *p* = 0.182 | 0.047 (0.142)  *p* = 0.742 | -0.120 (0.120)  *p* = 0.319 | | 0.066 (0.123)  *p* = 0.589 | -0.075 (0.174)  *p* = 0.665 | -0.119 (0.176)  *p* = 0.500 |
| Self-efficacy | -0.019 (0.072)  *p =* 0.789 | 0.004 (0.720)  *p* = 0.960 | -0.004 (0.014)  *p* = 0.791 | -0.004 (0.014)  *p* = 0.793 | -0.008 (0.013)  *p* = 0.521 | | 0.010 (0.014)  *p* = 0.469 | 0.006 (0.018)  *p* = 0.750 | 0.030 (0.018)  *p* = 0.092 |
| Nature connection | 0.342 (0.334)  *p =* 0.305 | -0.015 (0.342)  *p =* 0.964 | -0.067 (0.066)  *p =* 0.313 | 0.060 (0.068)  *p =* 0.378 | 0.018 (0.062)  *p =* 0.776 | | -0.068 (0.063)  *p =* 0.282 | -0.047 (0.082)  *p* = 0.564 | -0.045 (0.084)  *p* = 0.593 |
| Psychological benefits | 0.295 (0.289)  *p =* 0.308 | 0.331 (0.290)  *p =* 0.254 | -0.101 (0.057)  *p =* 0.079 | -0.029 (0.058)  *p =* 0.620 | -0.085 (0.054)  *p =* 0.114 | | -0.078 (0.054)  *p =* 0.152 | 0.012 (0.071)  *p =* 0.877 | 0.014 (0.071)  *p =* 0.845 |
| Awareness of global South | 0.293 (0.287)  *p =* 0.308 | 0.292 (0.298)  *p =* 0.328 | 0.019 (0.057)  *p =* 0.733 | 0.026 (0.059)  *p =* 0.664 | 0.086 (0.051)  *p =* 0.092 | | -0.018 (0.053)  *p =* 0.740 | 0.044 (0.070)  *p =* 0.530 | -0.060 (0.073)  *p =* 0.426 |
| Social norm ‘support’ | n/a | n/a | 0.003 (0.056)  *p =* 0.961 | -0.002 (0.056)  *p =* 0.976 | n/a | | n/a | 0.057 (0.069)  *p =* 0.411 | 0.129 (0.070)  *p =* 0.063 |
| Social norm ‘donation’ | n/a | n/a | n/a | n/a | -0.035 (0.053)  *p =* 0.514 | | 0.039 (0.060)  *p =* 0.512 | n/a | n/a |
| Climate change scepticism | -0.148 (0.224)  *p =* 0.508 | 0.020 (0.236)  *p =* 0.932 | -0.021 (0.046)  *p =* 0.629 | -0.153 (0.046)  ***p =* 0.001**, *p_FDR_ =* 0.006**** | -0.027 (0.042)  *p =* 0.527 | | -0.030 (0.046)  *p =* 0.514 | 0.026 (0.055)  *p =* 0.636 | -0.007 (0.058)  *p =* 0.900 |
| Flood experience | 0.403 (0.351)  *p =* 0.251 | 0.569 (0.350)  *p =* 0.104 | -0.045 (0.070)  *p =* 0.522 | -0.055 (0.069)  *p =* 0.425 | -0.034 (0.057)  *p =* 0.551 | | -0.039 (0.059)  *p =* 0.507 | 0.053 (0.086)  *p =* 0.538 | 0.046 (0.086)  *p =* 0.593 |

**Appendix S10:** Parameter estimates with standard errors (in parentheses) and *p*-values for interactions tests between dynamic norm-nudge and audience segmentation variables across the four outcome variables: advert sufficiency, sympathetic values, financial support, and behavioural support. Bold indicates significance. FDR adjusted p-values (*p_FDR_*) given for significant interactions.

| **Interaction (with norm-nudge)** | **Advert sufficiency** | **Sympathetic attitudes** | **Financial support** | **Behavioural support** |
| --- | --- | --- | --- | --- |
| Self-efficacy | -0.044 (0.059)  *p* = 0.454 | -0.002 (0.012)  *p* = 0.848 | -0.002 (0.011)  *p* = 0.827 | 0.009 (0.014)  *p* = 0.524 |
| Nature connection | 0.122 (0.279)  *p* = 0.663 | -0.022 (0.055)  *p* = 0.696 | 0.014 (0.052)  *p* = 0.783 | 0.017 (0.068)  *p* = 0.809 |
| Psychological benefits | 0.133 (0.242)  *p* = 0.584 | 0.072 (0.048)  *p* = 0.134 | 0.039 (0.045)  *p* = 0.388 | 0.147 (0.060)  ***p* = 0.013*,**  ***p_FDR_ =* 0.049*** |
| Awareness of global South | 0.155 (0.241)  *p* = 0.520 | 0.032 (0.049)  *p* = 0.508 | -0.055 (0.043)  *p* = 0.197 | -0.020 (0.059)  *p* = 0.736 |
| Social norm ‘support’ | n/a | -0.015 (0.047)  *p* = 0.750 | n/a | 0.024 (0.058)  *p* = 0.683 |
| Social norm ‘donation’ | n/a | n/a | 0.032 (0.046)  *p* = 0.485 | n/a |
| Climate change scepticism | 0.134 (0.190)  *p* = 0.480 | 0.004 (0.038)  *p* = 0.906 | -0.017 (0.036)  *p* = 0.630 | 0.071 (0.047)  *p* = 0.128 |
| Flood experience | 0.097 (0.299)  *p* = 0.745 | -0.032 (0.059)  *p* = 0.588 | -0.025 (0.046)  *p* = 0.579 | -0.008 (0.073)  *p* = 0.909 |
